# Epigenetic deprogramming by disruption of CIZ1-RNA nuclear assemblies in early-stage breast cancers

**DOI:** 10.1083/jcb.202409123

**Published:** 2025-03-11

**Authors:** Gabrielle L. Turvey, Ernesto López de Alba, Emma Stewart, Heather Cook, Ahmad Alalti, Richard T. Gawne, Justin F.-X. Ainscough, Andrew S. Mason, Dawn Coverley

**Affiliations:** 1 Mammalian Cell Cycle Research Group, Department of Biology, University of York, York, UK; 2 York Biomedical Research Institute, University of York, York, UK; 3 Jack Birch Unit for Molecular Carcinogenesis, Department of Biology, University of York, York, UK

## Abstract

CIZ1 is part of the RNA-dependent supramolecular assemblies that form around the inactive X-chromosome (Xi) in female cells and smaller assemblies throughout the nucleus in both sexes. Here, we show that CIZ1 C-terminal anchor domain (AD) is elevated in human breast tumor transcriptomes, even at stage I. Elevation correlates with deprotection of chromatin and upregulation of lncRNA-containing gene clusters in ∼10 Mb regions enriched in cancer-associated genes. We modeled the effect of AD on endogenous CIZ1–Xi assemblies and observed dominant-negative interference with their reformation after mitosis, leading to abnormal assemblies similar to those in breast cancer cells, and depletion of H2AK119ub1, H3K27me3, and *Xist*. Consistent alterations in gene expression were evident across the genome, showing that AD-mediated interference has a destabilizing effect, likely by unscheduled exposure of underlying chromatin to modifying enzymes. The data argue for a dominant, potent, and rapid effect of CIZ1 AD that can deprogram gene expression patterns and which may predispose incipient tumors to epigenetic instability.

## Teaser

This paper shows that protein fragments derived from the CIZ1 gene disperse nuclear CIZ1-RNA assemblies, exposing underly loci and changing gene expression. The interference mechanism is implicated as a driver of epigenetic instability in early-stage breast cancers.

## Introduction

Selection and packaging of chromatin into transcriptionally repressed states underlie cell specialization and development. Weakened repression of heterochromatin can result in pro-oncogenic changes and has the potential to give rise to all the classic hallmarks of cancer, even in the absence of genetic change (Flavahan et al., 2017; Hanahan, 2022; Parreno et al., 2024). The inactive X chromosome (Xi) is the most intensely studied model of facultative heterochromatin formation, revealing how the *cis*-acting lncRNA *Xist* (Brockdorff et al., 1992; Brown et al., 1992) directs the formation of large RNA-dependent supramolecular assembly complexes (SMACs) populated by chromatin-modifying enzymes (Markaki et al., 2021). Aggregation of SMAC proteins, mediated by their intrinsically disordered regions (IDRs), creates a functional nuclear compartment that partitions regulatory factors to establish local gene silencing early in development.

Cip1-interacting zinc finger protein 1 (CIZ1) is one of several proteins that populate Xi SMACs, recruited via its interaction with the repeat E element of *Xist* (Ridings-Figueroa et al., 2017; Sunwoo et al., 2017). Several observations set CIZ1 apart from other SMAC components. First, it is not required for *Xist* recruitment, Xi silencing, or embryonic development, and the impact of its loss only becomes apparent in somatic cells in which repressed chromatin is already established but must be faithfully maintained. A requirement for CIZ1 is apparent in differentiated fibroblasts from CIZ1 null mice, where local retention of *Xist* around Xi chromatin is compromised (Ridings-Figueroa et al., 2017; Sunwoo et al., 2017), repressive histone posttranslational modifications (PTMs) are lost, and genome-wide changes in the expression of genes under the regulation of polycomb repressive complexes (PRC 1 and 2) are apparent (Stewart et al., 2019). Second, the stability of CIZ1 within Xi SMACs, even those that form during the initiation stages of X-inactivation, is unusually high. Compared with other SMAC components, the residency time of CIZ1 is estimated to be 2–10-fold longer, similar to that of *Xist* (Markaki et al., 2021). Thus, it appears that CIZ1 exchanges less readily than other protein components and might therefore contribute a stabilizing influence on *Xist* and Xi SMACs.

Some of the sequence determinants required for the assembly of CIZ1 within Xi SMACs are known, including two alternatively spliced, low-complexity prion-like domains (PLD1 and PLD2) that modulate interaction with *Xist*, and a second RNA interaction domain in the C-terminus (Sofi et al., 2022). Neither RNA interaction is sufficient to support the assembly of CIZ1 into Xi SMACs on its own, but together, they drive both the assembly and the de novo enrichment of H2AK119ub1 and H3K27me3, added by PRC1 and 2, respectively, in the underlying chromatin. These experiments directly link CIZ1 SMAC formation with the modification of chromatin and implicate its bivalent interaction with RNA (Sofi et al., 2022).

Disappearance of the Barr body (Xi) has been known for decades and is considered a hallmark of cancer (Moore and Barr, 1957). Erosion of the Xi in breast tumors and cell lines was originally ascribed to genetic instability, though epigenetic instability is also apparent, evident as an abnormal subnuclear organization, aberrant promoter DNA methylation, and perturbations of chromatin, including H3K27me3 (Chaligné et al., 2015). Transcriptional reactivation of X-linked genes has been implicated in both breast and ovarian cancers (Sirchia et al., 2009) though is likely to be indicative of wider, and possibly earlier, epigenetic erosion. In fact, widespread erosion of the DNA methylation landscape can give rise to the transcriptional changes common in tumors (Batra et al., 2021), and for breast cancers in particular, the progression from progenitor cell to premalignant lesion has been shown to involve changes in the DNA methylome that precede genetic instability (Locke and Clark, 2012; Locke et al., 2015). From data such as these, a model is emerging in which induction of breast cancer could occur primarily through epigenetic disruption.

Here, we describe the aberrant expression of CIZ1 in human cancers and model the effects of destabilizing protein fragments on RNA–protein assemblies and underlying chromatin. The data lead to the conclusion that disease-associated dominant-negative CIZ1 fragments (DNFs) contribute to epigenetic instability by deprotecting loci that are normally buffered by surrounding SMACs and that this plays an early role in tumor etiology by promoting epigenetic instability.

## Results

### CIZ1 assemblies are disrupted in breast cancer cells

**Figure 1.**
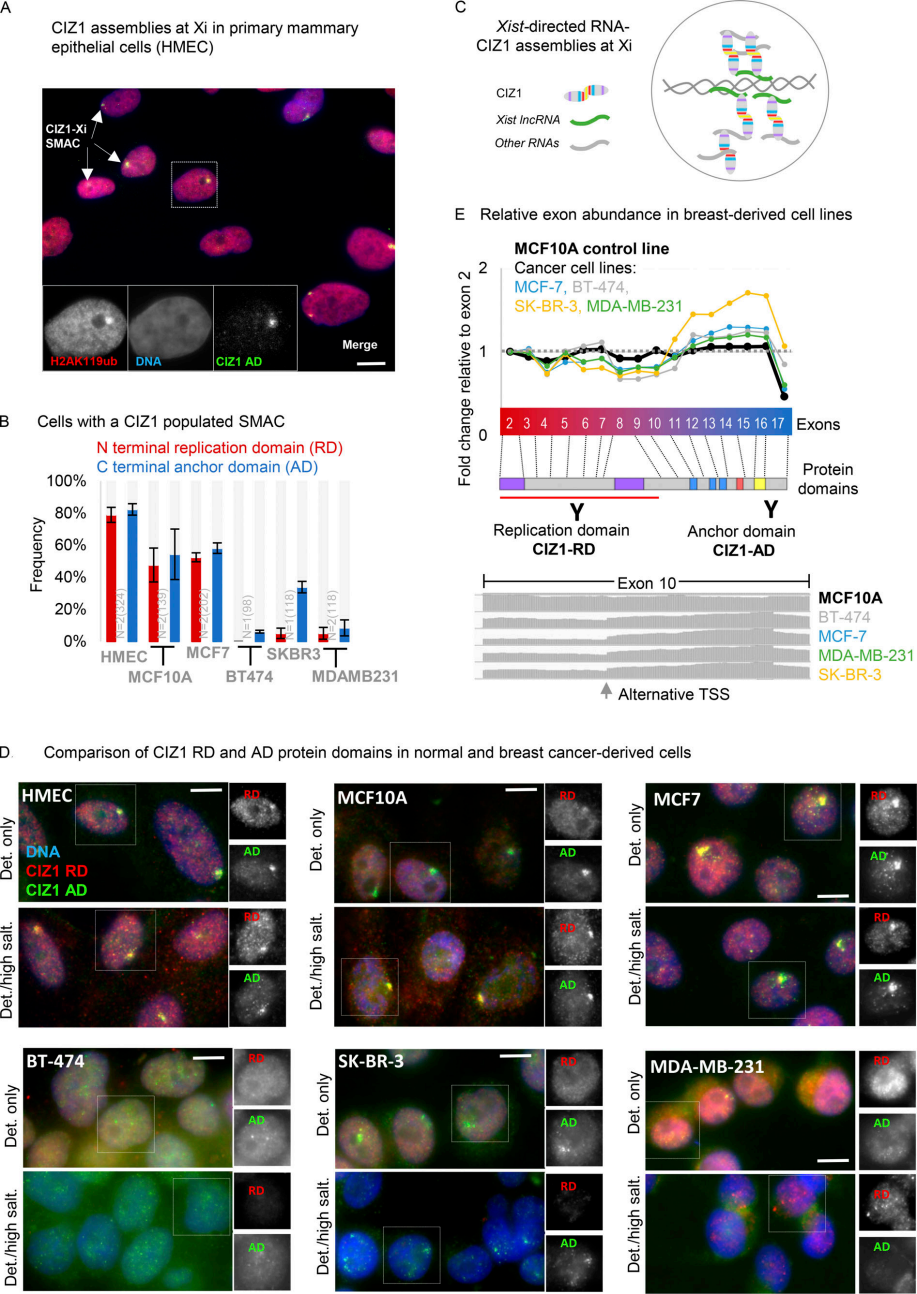
**Corrupted CIZ1–Xi assemblies in breast cancer cells. (A)** Female primary human breast epithelial cells (HMECs) stained for CIZ1 via its C-terminal anchor domain (AD, green), and co-localization with H2AK119ub1 (red) as a marker of Xi chromatin. DNA is blue. Inset, example nucleus with CIZ1–AD and H2AK119ub1 shown individually in grayscale. Bar is 10 μm. **(B)** Frequency of cells with discrete nuclear CIZ1 assemblies, detected via CIZ1-AD (blue) or CIZ1-RD (red) in cycling populations of the indicated breast-derived cell types. Error bars show SEM. A reduced frequency of CIZ1–Xi assemblies is observed in non-cancer breast cell line MCF-10A and cancer cell line MCF7, while in the more aggressive BT-474 and MDA-MB-231 cancer cells, large CIZ1 SMACs are rare for both RD and AD epitopes, and in SK-BR-3 populations only detectable via the AD epitope. In all four of the cancer lines, the appearance of those assemblies that are detected is less compact and coherent (see part D). **(C)** Model showing multivalent interaction between CIZ1 N- and C-terminal RNA interaction domains, and RNAs including *Xist* in the vicinity of the inactive X chromosome (Sofi et al., 2022). **(D)** Example immunofluorescence images of CIZ1-RD (red) and CIZ1-AD (green) in HMEC and the indicated breast cancer cell lines, after pre-fixation wash with detergent-containing buffer (Det. only), or after high-salt extraction (Det./high salt). Right, nuclei in which RD and AD are shown individually in grayscale. Bar is 10 μm. The RD and AD epitopes were differentially detected or extracted in some cases, indicating that they are not always part of the same polypeptide (for example compare nucleus-wide RD in SK-BR-3 cells, in detergent-treated cells to detergent/high-salt treated cells). **(E)** CIZ1 exon-specific TPMs from four breast cancer (MCF7, BT-474, SK-BR-3, and MDA-MB-231) and one normal breast-tissue derived cell model (MCF10A), normalized to the first translated exon (exon 2), showing imbalanced domain expression, favoring the C-terminal anchor domain (AD). Exon map is aligned with protein domains (see also Fig. S1 A), and the location of epitopes used to report on CIZ1-AD (green, Ab87) or CIZ1 replication domain (RD, red, Ab1793) are shown. Below, the relative frequency of reads aligning to human CIZ1 exon 10, demonstrating consistent coverage in the normal MCF10A line, and a transition in the cancer cell lines within exon 10, at the location of an alternative transcription start site (see also Fig. S1 C).

**Figure S1.**
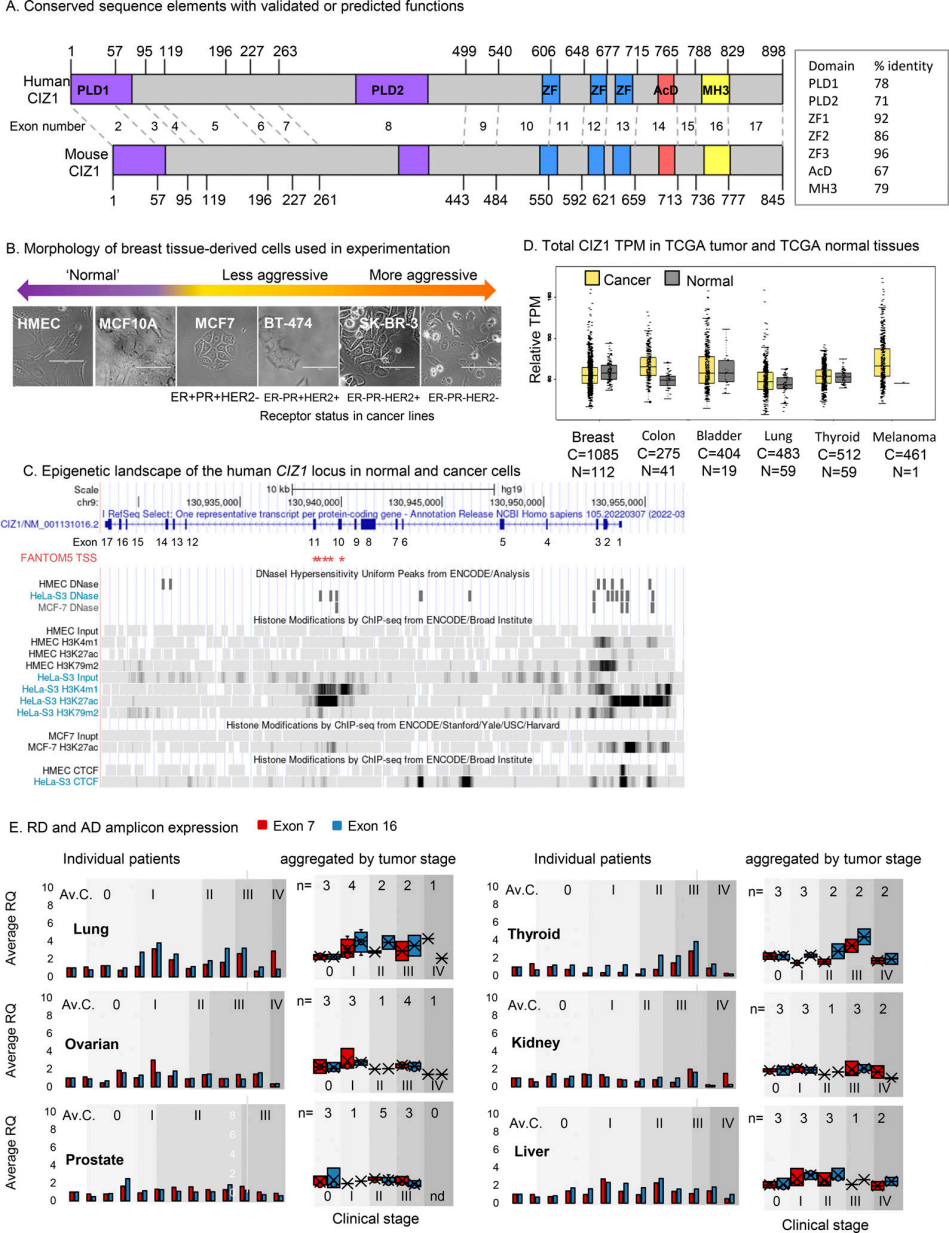
**(Related to** Fig. 1 **and** Fig. 2 **). CIZ1 domains, transcript levels**, **and domain expression in common solid tumors. (A)** Protein domain map aligning human (NP_001124488.1) and mouse (NP_082688.1) CIZ1. Numbers correspond to amino acids encoded at exon boundaries. The domains highlighted are: Prion-like domains 1 and 2 (PLD1 and PLD2, purple) at positions 1–78 and 360–451, respectively (human), and positions 1–67 and 361–399, respectively (mouse),^10^ three zinc fingers (ZnF_C2H2 SM00355, ZF_C2H2 sd00020, and ZF_C2H2 sd00020, blue) at positions 593–617, 656–676, and 687–709, respectively (human), and 537–561, 600–620, and 631–653, respectively (mouse), an acidic domain (red) containing a concentrated area of aspartates and glutamates at position 741–761 (human) and 689–709 (mouse), and a matrin-3 homology domain (ZnF_U1 smart0045, yellow) at position 796–831 (human) and 746–770 (mouse). Box shows % identity at the amino acid level across these domains. Human and mouse CIZ1 are 65% identical at the protein level, with identity concentrated in the conserved domains (up to 96%). **(B)** Bright-field images of breast-derived cell types ordered based on phenotype, with corresponding hormone and growth factor receptor status. The bar is 100 μm. **(C)***CIZ1* locus in *Homo sapiens* with corresponding exon numbers. Potential *CIZ1* alternative transcription start sites (TSSs) in exons 10 and 11 predicted in the FANTOM5 project (Lizio et al., 2015) are indicated (red stars). The coding sequence would be expected to begin at a methionine in exon 11. The chromatin landscape in human mammary epithelial cells (HMEC), a cervical cancer cell line (HeLa) and a breast cancer cell line (MCF7) is shown below. Diagram generated using UCSC genome browser (Cunningham et al., 2022). **(D)** Total CIZ1 TPM derived from the indicated number of cancer (C) and normal (N) tissues in TCGA compared using GEPIA for the indicated disease types. No significant difference is detected (where log_2_FC was >1, and P value <0.05) when comparing all amalgamated transcripts that map to the CIZ1 gene (unresolved by exon). **(E)** Relative expression of exons 7 (red) and 16 (blue), normalized to the average of three unmatched control samples for each of six common solid tumor types in multi-tissue cDNA array CSRT101. Individual patient data plus the average of the controls calibrated to 1 (Av.C, left) and data aggregated by disease stage (0–IV, right) are shown. 0 represents histologically normal tissue. Individual sample information for all arrays is given in Data S2.

In primary epithelial cells derived from normal human female mammary tissue (HMECs), a single large CIZ1 assembly is visible in ∼80% of cells in a cycling population (Fig. 1, A and B). This coincides with local enrichment of H2AK119ub1 identifying the assembly as at the Xi, as reported for humans (Dixon-McDougall and Brown, 2022; Ridings-Figueroa et al., 2017; Valledor et al., 2023) and murine cells (Markaki et al., 2021; Ridings-Figueroa et al., 2017; Sunwoo et al., 2017). Human and murine CIZ1 possess the same conserved domains encoded by the same exons in the same order (Fig. S1 A), and so far no differences in the behavior or function have been uncovered. CIZ1–Xi assemblies are dependent on multivalent interaction with RNAs including *Xist* (Fig. 1 C [Sofi et al., 2022]) and normally observed with similar frequency regardless of whether epitopes in its N-terminal DNA replication domain (RD) (Coverley et al., 2005) or C-terminal nuclear matrix anchor domain (AD) (Ainscough et al., 2007) are detected (Fig. 1 B).

However, in breast cancer–derived cell lines (Fig. S1 B), the same anti-CIZ1 RD and anti-CIZ1 AD antibodies reveal considerable heterogeneity. CIZ1 Xi assemblies are either absent, less compact, and coherent, or RD and AD epitopes are differentially susceptible to extraction from the nucleus (Fig. 1, B and D). This indicates that CIZ1 RD and AD are not always part of the same polypeptide and are compromised in their ability to form stable assemblies around Xi chromatin. We conclude that CIZ1 protein and CIZ1–Xi assemblies are commonly disrupted in breast cancer cell lines. This is consistent with the reported wider destabilization of the inactive X chromosome in breast cancer cells and tissues and specifically the reported dispersal of *Xist* (Chaligné et al., 2015).

Alignment of transcriptomes from four breast cancer-derived cell lines and a control cell line to CIZ1’s translated exons (2–17) revealed over-representation of AD-encoding exons in the tumor-derived lines compared with RD-encoding exons (Fig. 1 E and Data S1). We also noted a transition in transcript coverage within exon 10, which coincides with an internal transcription start sites (TSS) annotated in Ensembl (Cunningham et al., 2022) from the FANTOM5 project (Lizio et al., 2015), and with enrichment of indicators of active chromatin in cancer cell lines but not normal HMECs (Fig. S1 C). Thus, archive data suggest that transcription can begin from an internal site in the CIZ1 gene.

### Elevation of CIZ1 AD-encoding transcript in early-stage primary breast tumors

**Figure 2.**
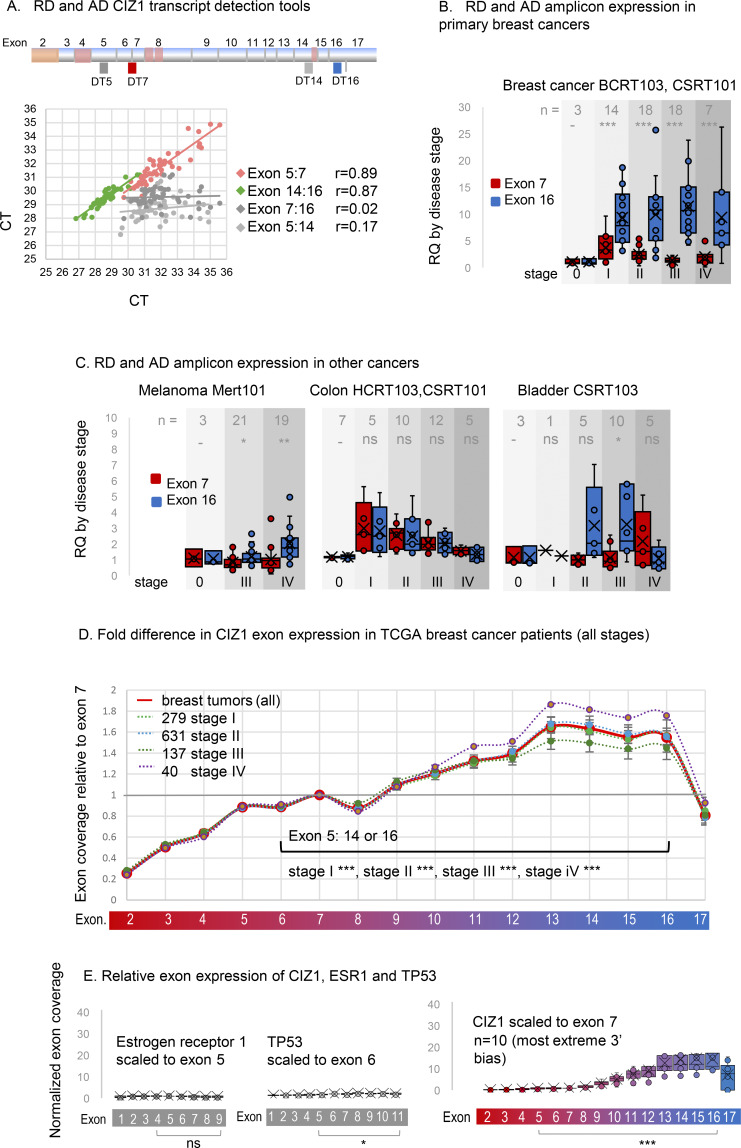
**Elevated CIZ1 anchor domain expression in primary cancers. (A)** Exon structure of CIZ1 based on human reference sequence NM_012127.2 showing all 16 translated exons (2–17), and those subject to alternative splicing (pink) (Coverley et al., 2005; Dahmcke et al., 2008; Higgins et al., 2012; Rahman et al., 2007; Sofi et al., 2022; Swarts et al., 2018). Alternative untranslated exons 1’s are not shown. The location of amplicons detected by quantitative RT-PCR detection tools (four Taqman primer/probe sets; DT5 and DT7 which detect the 5′ end of CIZ1 transcripts, and DT14 and DT16 which detect the 3′ end) are indicated. Below, the dot plot shows the comparison of outputs with the indicated pairs applied to 46 human tissue-derived cDNAs. Pearson’s correlation coefficients show strong agreement between exons 5 and 7, and between 14 and 16, but poor agreement between exons 7 and 16, or 5 and 14, indicating that the 5′ and -3′ ends of CIZ1 are typically imbalanced at the transcript level. **(B)** Relative quantification (RQ) of CIZ1 exon 7 (red) and CIZ1 exon 16 (blue) in primary human breast tissue-derived cDNAs in arrays BCRT103 and CSRT101 (*n* = 60, all female). Box and whisker plots show results aggregated by clinical stage (0–IV), calibrated to the average of the stage 0 samples for each amplicon where 0 represents histologically normal tissue. Significance indicators show comparisons between amplicons by *t* test, where ns is not significant, *P < 0.05, **P < 0.01, ***P < 0.001. Individual sample values are given in Data S2. **(C)** As in B for human tissue-derived cDNAs in arrays MERT101 (melanoma, *n* = 43), HCRT103, CSRT101 (colon, *n* = 39), and CSRT103 (bladder, *n* = 24). **(D)** CIZ1 exon expression in TCGA breast cancer samples, separated by clinical stage and normalized to individual exon 7 expression. At all stages, 5′ and 3′ expression is significantly different, with 3′ elevation from around exon 10. Comparison of transcript levels in exon 5 to 14 or 16 (arrows) is by Mann–Whitney U test. Error bars show SEM. *n* = 1,087, 99% female. **(E)** Control analysis showing TPMs in a subset of 10 stage 2 TCGA breast cancer patients that exhibit the most marked 3′ end bias for CIZ1, mapped to CIZ1 exons, normalized to exon 7. Left, TPMs from the same patients for estrogen receptor alpha (ERα/ESR1) normalized to its exon 5, and TP53 normalized to its exon 6, showing relative exon coverage and lack of 3′ over-representation.

To measure CIZ1 transcript expression in primary common solid tumors, we first used quantitative RT-PCR to compare the 5′ end to the 3′ end (which contribute coding sequence to RD and AD respectively) by detection of amplicons unaffected by alternative splicing (Rahman et al., 2010) (Fig. 2 A). In cDNAs from 46 tissue samples, the correlation between two RD amplicons (in exons 5 and 7) or between two AD amplicons (in exons 14 and 16) was strong; however, RD and AD did not correlate with each other. This confirms that expression of RD and AD are commonly uncoupled at the transcript level and shows that the differential can be sampled by comparing sequences in the region of exons 5–7 to sequences in the region of exons 14–16.

Domain disparity was striking and consistent in breast tumors across all stages (Fig. 2 B). It was also significant in bladder cancer at stage III and melanoma at stages III and IV (Fig. 2 C) and observed sporadically in other tumors of different etiology (Fig. S1 E). In addition, in some colon, lung, and thyroid tumors both RD and AD domains of CIZ1 were elevated compared with histologically normal tissue (Fig. 2 C, Fig. S1 E, and Data S2).

Focusing on breast cancer, we analyzed *CIZ1* expression in 1,095 transcriptomes submitted to The Cancer Genome Atlas (TCGA). While transcripts that map to the whole *CIZ1* gene revealed no overall difference in expression between tumors and normal tissue (Fig. S1 D), the same raw data when mapped to individual CIZ1 exons showed that AD (exon 14) is significantly over-represented compared with RD (exon 5) at all stages (Fig. 2 D and Data S1) and that AD elevation was notable from around exon 10. Similar elevation of C-terminal transcript was not evident in cancer-associated genes ESR1 and TP53 in a subset of the same transcriptomes (Fig. 2 E). Together, these data show that C-terminal CIZ1 exons are over-represented in the majority of breast cancers, that epitopes encoded by C-terminal exons are uncoupled from N-terminal exons and pose the question of whether inappropriate AD protein is functionally relevant.

### In vitro modeling of the effect of AD on CIZ1–Xi assemblies

**Figure 3.**
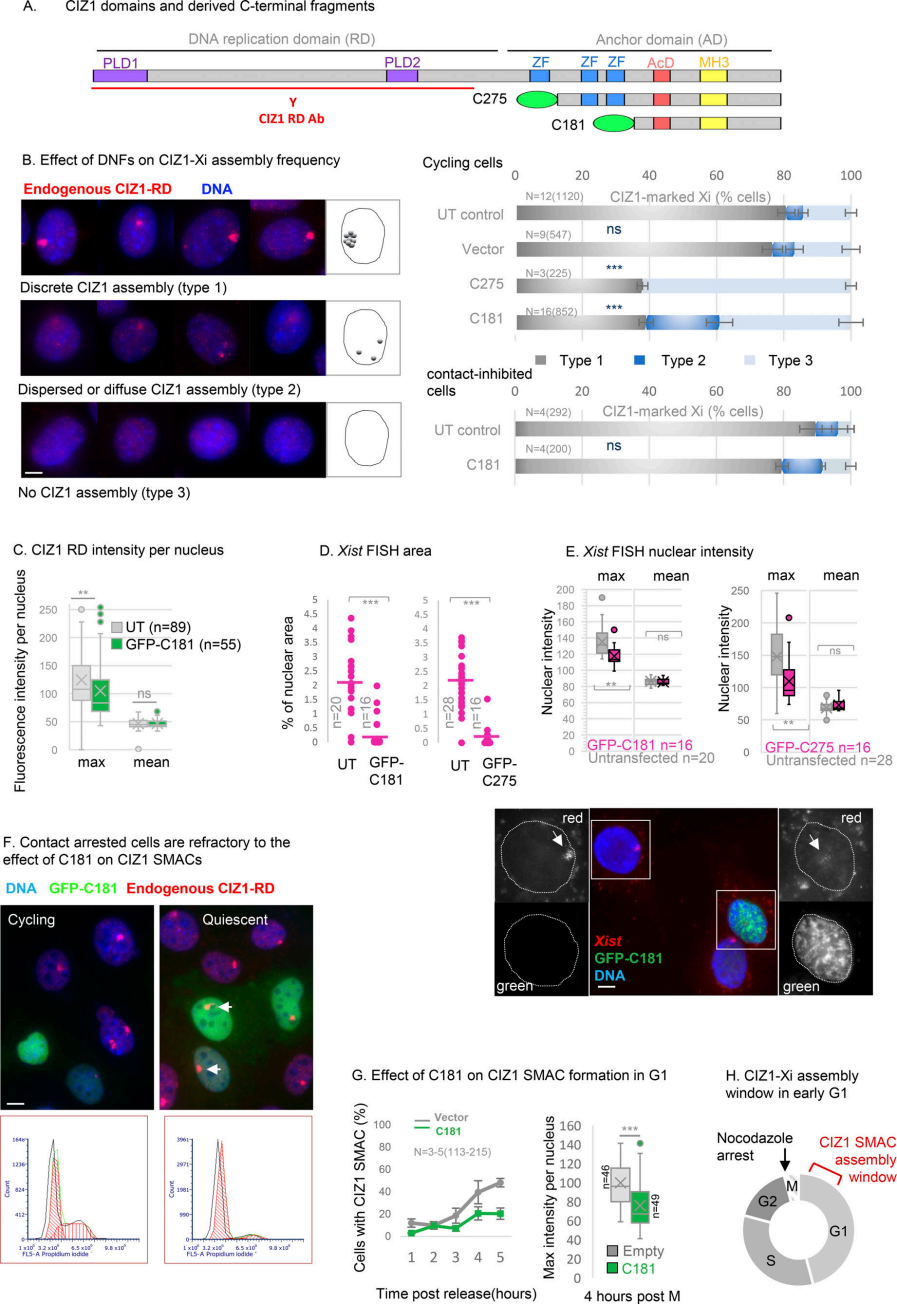
**Dispersal of endogenous CIZ1 Xi SMACs by ectopic CIZ1 anchor domain. (A)** Diagram of murine CIZ1 full-length protein showing prion-like domains (PLD) (Sofi et al., 2022), zinc fingers (ZF), acidic domain (AcD), and Matrin 3 homology domain (MH3, see also Fig. S1 A). Below, C-terminal protein fragments C275 and C181 correspond to the terminal 275 and 181 amino-acids respectively, both bearing an N terminal GFP tag. CIZ1 RD antibody (red) was raised against a fragment of CIZ1 outside of C275 (Coverley et al., 2005) and does not detect the transgenes. **(B)** Left, example images of CIZ1–Xi assemblies in D3T3 cells, showing three categories of nuclei with either large discrete CIZ1 SMACs (type 1, upper), no detectable CIZ1 SMAC (type 3, lower), or intermediate assemblies (type 2, middle), which include those that are either dispersed into multiple smaller foci or diminished in overall size or intensity. CIZ1 is red, DNA is blue. Right, frequency of cells with type 1, 2, or 3 CIZ1 Xi assemblies in untransfected (UT) populations, compared to those expressing empty GFP vector, C275 or C181. N is replicate analysis, with total nuclei inspected shown in parentheses. Error bars show SEM. For cycling cells (upper), no difference in frequencies was observed between untransfected (UT) and empty vector cells (type 1 P = 0.72, type 2 P = 0.19, type 3 P = 0.75), but dispersal was observed in C181 expressing cells compared with UT (type 1 P = 1.4 × 10^−5^, type 2 P = 0.0036, type 3 P = 0.00032). For contacted cells (lower), none or limited differences in frequencies were observed between UT and C181 expressing cells (type 1 P = 0.098, type 2 P = 0.34, type 3 P = 0.043). All comparisons of replicate analyses are by unpaired *t* test. **(C)** Box and whisker plots showing CIZ1-RD fluorescence intensity per nucleus in untransfected (UT, gray) and C181 transfected (green) WT female PEFs, showing no difference in means but a significant reduction in maxima. *n* is nuclei measured, comparison by *T* test. **(D)** Area occupied by *Xist* FISH, calculated as % of nuclear area delineated by DAPI stain, in cells expressing GFP-C181 or C275, and untransfected cells (UT) in the same populations. *n* is nuclei measured, comparisons by *T* test. **(E)** The intensity of nuclear *Xist* FISH signal, in the same cells as D, showing intensity maximas and means as box and whisker plots. Below are example images (see also Fig. S2 A) showing *Xist* (red) in D3T3 cells with and without expression of GFP-C181 for 24 h. Insets illustrate compact *Xist* in an untransfected cell and dispersed *Xist* in a transfected cell (green). DNA is blue in the main image and used to create nuclear outlines (dotted lines) in insets, the bar is 5 μm. **(F)** Field images showing untransfected and transfected cycling and contact inhibited D3T3 cells, illustrating the effect on endogenous CIZ1 status at Xi (red). Arrows point to resistant CIZ1 Xi assemblies in contact-inhibited cells. Below are flow cytometry profiles of populations stained with propidium iodide, illustrating G1/G0 enrichment in the contacted cell population. **(G)** Impaired reformation of CIZ1 SMACs after release from arrest in M phase in D3T3 cells transduced with C181 compared with vector control. Left, CIZ1–Xi assembly frequency 1–5 h after release. Right, box and whisker plot showing maximum fluorescence intensity per nucleus at 4 h, where *n* indicates the number of nuclei measured in each group. Mann–Whitney U test, P = 6.1 × 10^−6^. **(H)** Illustration showing the time window of CIZ1 Xi SMAC assembly early in the G1 phase and the point of cell cycle arrest after exposure to nocodazole.

We previously showed that ectopic full-length CIZ1 accumulates within CIZ1–Xi assemblies in WT cells and can in fact build new assemblies de novo in CIZ1 null cells, provided both RD and AD are present (Sofi et al., 2022). The multivalent nature of CIZ1’s interaction with RNA and the requirement for both domains for assembly into SMACs (Sofi et al., 2022), lead us to hypothesize that fragments of CIZ1 encoding only one of its RNA interaction interfaces might have a destabilizing effect. Moreover, based on what we know of CIZ1 genetic deletion and the co-dependency of CIZ1 and *Xist* (Markaki et al., 2021; Ridings-Figueroa et al., 2017; Rodermund et al., 2021; Sunwoo et al., 2017), we hypothesized that interference with CIZ1 assemblies at Xi would affect Xi chromatin. We modeled this in short-term (one-cell cycle) transfection experiments after ectopic expression of GFP-tagged C-terminal protein fragments (Fig. 3 A) in murine cells.

**Figure S2.**
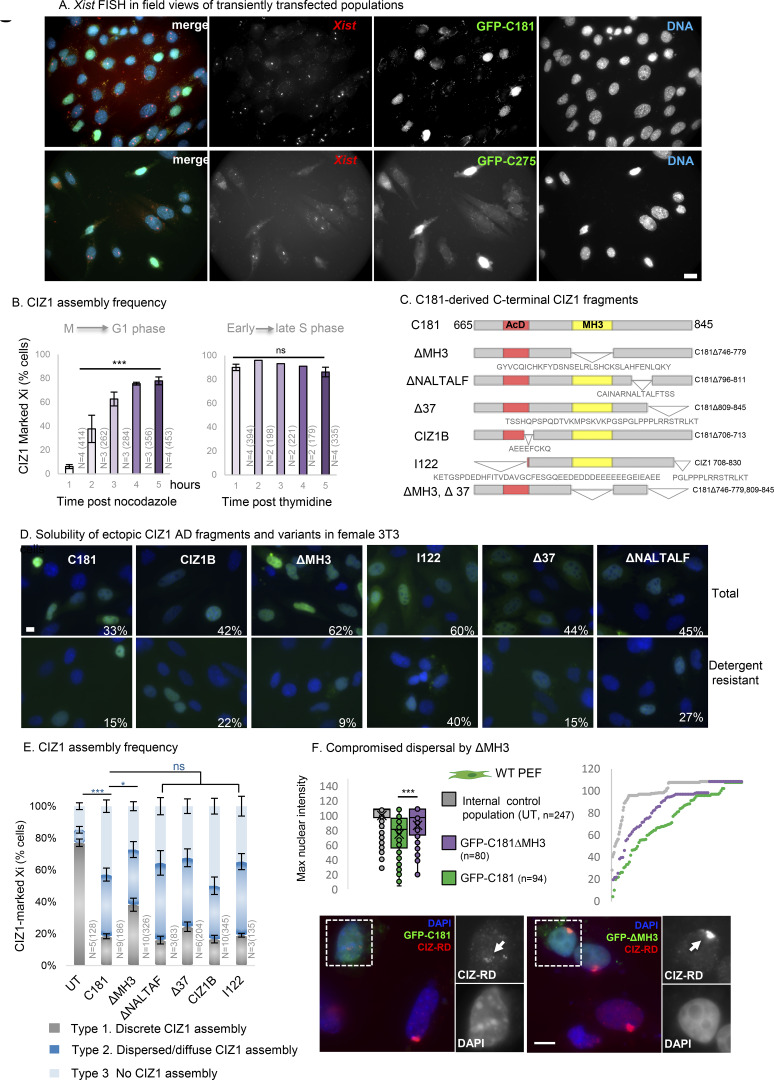
**(Related to** Fig. 3 **). Cell cycle analysis and anchor domain mutagenesis. (A)** Field views of *Xist* (red) in cycling D3T3 cells, 24 h after transfection with GFP-C181 or C275 (green) as indicated. DNA is blue. The bar is 10 μm. **(B)** CIZ1–Xi assembly frequency 1–5 h after release from cell cycle arrest in S phase (thymidine) or M phase (nocodazole). *N* is 2–4 as indicated, number of nuclei inspected at each time point is given (*n*). Comparisons between 1 and 5 h *t* test, where P = 0.45 for S phase and P = 1.15 × 10^−6^ for M–G1 phase. **(C)** Map of C181 deletion constructs, showing excluded sequences in single letter code. These exclude the MH3 domain (conserved domain ZnF_U1 smart00451) or the fully human/mouse conserved sequence downstream of the MH3 domain (ΔNALTALF) or the murine equivalent of the eight amino acids previously implicated in lung cancer (CIZ1B) (Higgins et al., 2012), or the terminal 37 amino acids (Δ37). We also evaluated a fragment encompassing the MH3 domain but lacking sequences up and downstream (I122) (Ainscough et al., 2007). Numbers indicate amino acid at boundaries relative to murine full-length CIZ1. AcD, acidic domain (red), and MH3, matrin 3 homology domain (yellow). **(D)** Example images of D3T3 cells expressing GFP-tagged C181-derived deletion mutants, without (total) and with (detergent-resistant) prefixation wash with 0.05% Triton X-100. The percentages show the proportion of transfected cells in each population with nuclear C181 or derivative, revealing the degree of sensitivity to extraction. The bar is 5 μm. **(E)** Effect of fragments on the frequency of endogenous CIZ1–Xi assemblies in D3T3 cells. Compared with C181, only ΔMH3 was perturbed in its ability to disperse endogenous CIZ1 (P = 0.011). All other deletion mutants retained similar DNF capability to C181 (ΔNALTALF P = 0.96, Δ37 P = 0.64, CIZ1B P = 0.99, I122 P = 1). N shows replicate analyses with total nuclei inspected in parentheses. Comparisons are by one-way ANOVA. Error bars show SEM. **(F)** Left, box and whisker plot showing normalized endogenous CIZ1-RD fluorescence intensity per nucleus in female WT PEFs, either untransfected (UT) or with C181, or derived deletion mutant ΔMH3, showing reduced potency of ΔMH3 compared to C181 (P = 0.002, *t* test). Right, mean intensity measures are ordered low to high for endogenous CIZ1 in UT, C181, and ΔMH3 expressing cells. Below are example images of cells stained for endogenous CIZ1 (red), with and without ectopic GFP-C181 or GFP-ΔMH3. Bar is 5 μm. The inset shows surviving Xi assemblies in grayscale.

Endogenous CIZ1–Xi assemblies were categorized into three phenotypes: cells with a discrete normal assembly, cells with no assembly, or cells with intermediate, dispersed, or diminished assemblies (Fig. 3 B). The C-terminal 275 amino-acids of murine CIZ1 (Coverley et al., 2005), here referred to as C275, caused loss or reduction in normal (type 1) assemblies but, as reported previously (Sofi et al., 2022), did not itself accumulate at Xi. In untransfected cells in the same populations or parallel populations expressing empty GFP vector, CIZ1–Xi assemblies were unaffected, all evidenced via detection of CIZ1 RD epitope (not present in C275). Notably, the deletion of two zinc fingers in C275 to produce the smaller C181 fragment did not abolish the disruptive effect on assembly frequency (Fig. 3 B), confirmed by measuring maximum fluorescence intensity per nucleus as a surrogate for CIZ1 assembly density (Fig. 3 C, left). A concomitant effect on *Xist* was confirmed by RNA FISH for both C275 and C181 (Fig. S2 A) by quantifying either the area occupied by *Xist* assemblies (Fig. 3 D) or the maximum fluorescence intensity per nucleus (Fig. 3 E). Notably, for both CIZ1 (Fig. 3 C, right) and *Xist* (Fig. 3 E), the mean intensity per nucleus remains unaffected, suggesting that while their ability to accumulate in Xi-associated assemblies is impaired, their overall levels in the nucleus remain the same. Thus, the data show that C-terminal fragments of CIZ1 do have the capacity to interfere with endogenous CIZ1–Xi assemblies, driving dispersal of both endogenous CIZ1 and *Xist* lncRNA, and are referred to hereafter as CIZ1 DNFs (dominant-negative fragments).

### CIZ1 assembly dispersal is cell cycle-dependent

Not all cells expressing CIZ1 DNFs are depleted of endogenous CIZ1–Xi assemblies. At 24 h, typically 30–40% remain refractory (Fig. 3 B), and in those that respond, the extent of dispersal is variable. We tested whether the cell cycle stage contributes to the heterogenous response initially by testing contact-inhibited (arrested) cells (Fig. 3 F). Under these conditions, CIZ1–Xi assemblies were refractory to the dominant negative effects of C181 (Fig. 3 B), suggesting that passage through the cell cycle is required to expose assemblies to a window in which DNFs can exert their effect.

Normally around 80% of female cells (cycling, mouse or human, primary or established non-cancer lines) contain a discrete compact CIZ1–Xi assembly. Since we know that, like *Xist* (Hall et al., 2009), CIZ1–Xi assemblies are lost in mitosis (Ridings-Figueroa et al., 2017) we postulated that those cells in which they are not evident have yet to rebuild them and are in early G1 phase. We confirmed this in cells synchronized in mitosis using nocodazole and found that maximal CIZ1–Xi assembly frequency was reached by 4 h after mitotic exit (Fig. S2 B). Expression of C181 significantly delayed SMAC reformation during this window and those that did form had reduced CIZ1 maximum fluorescence intensity (Fig. 3 G). Thus, the dispersive effect of CIZ1 DNFs is potent during the SMAC assembly window in the early G1 phase (Fig. 3 H).

### Role of the MH3 homology domain

To refine the sequence requirements for SMAC dispersal by DNFs, we evaluated a set of six deletion constructs based on C181 (Fig. S2 C). All fragments were expressed and became incorporated into detergent-resistant nuclear structures (Fig. S2 D) and retained similar capability to interfere with endogenous CIZ1–Xi SMACs, with the exception of one. C181 lacking the Matrin 3 homology domain (ΔMH3) had a small but consistent reduction in potency based on SMAC frequency (Fig. S2 E), confirmed by measuring maximum fluorescence intensity (Fig. S2 F). This implicates the MH3 CIZ1:CIZ1 dimerization interface (Turvey et al., 2023, *Preprint*) in the integrity of endogenous CIZ1 SMACs.

### Consequences of dispersal of CIZ1–Xi assemblies on Xi chromatin

**Figure 4.**
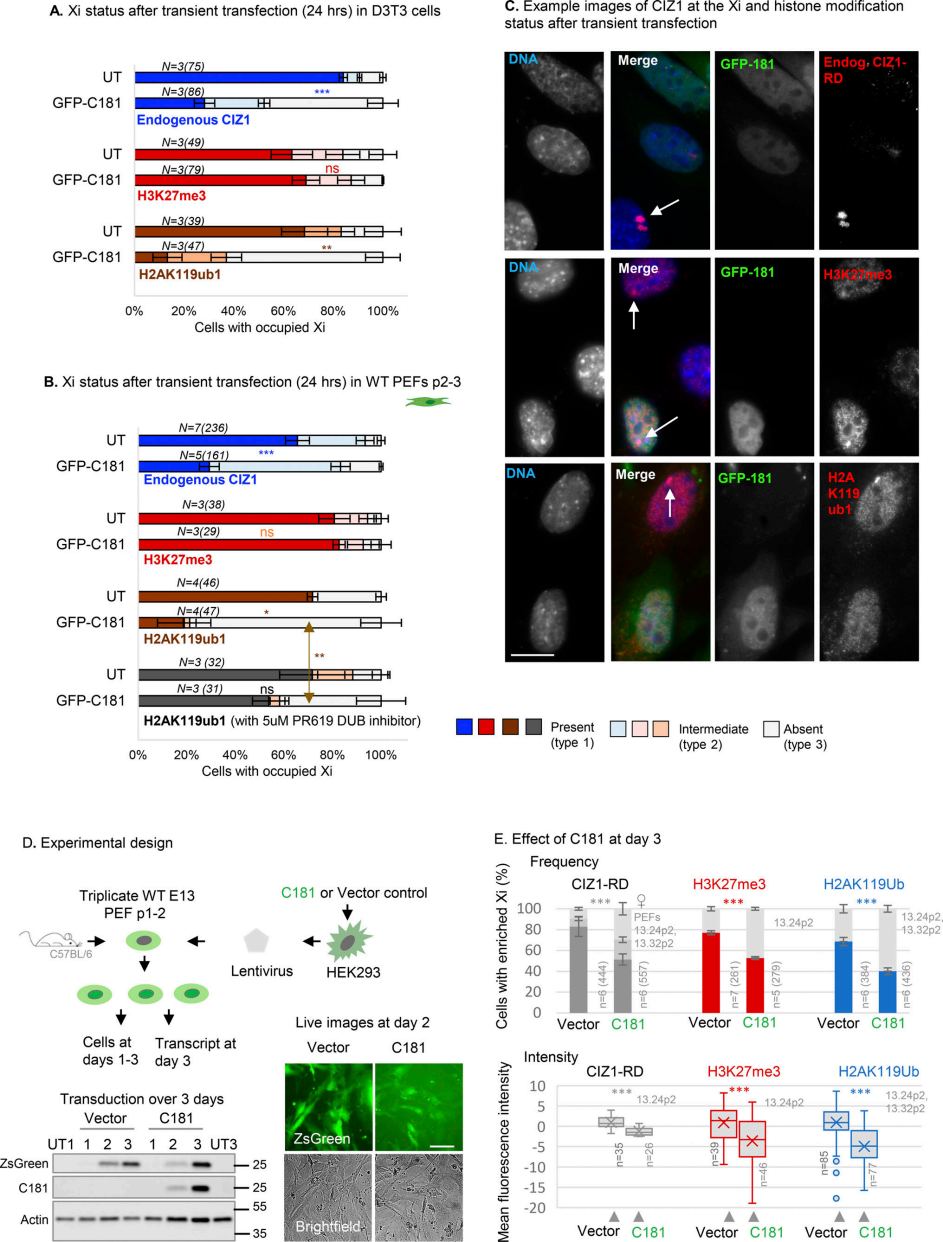
**Effect of CIZ1 anchor domain on histone posttranslational modifications. (A)** Graphs show the frequency of endogenous CIZ1–Xi assemblies in a cycling population of female D3T3 cells, comparing transfected and untransfected cells in the same population. Endogenous CIZ1 assemblies are detected via CIZ1-RD and classified into three categories; present, absent or intermediate. Middle and lower graphs show the frequency of repressive histone marks in cells that are, or are not transfected with GFP-C181. *N* is replicate analyses with nuclei scored in parentheses. Comparisons are by *t* test. For endogenous CIZ1 in UT and C181 cells P = 0.00023, for H3K27me3 P = 0.60, for H2AK119ub1 P = 0.0073. Error bars show SEM. **(B)** As in A, except that all data is derived from analysis of female primary embryonic fibroblasts (PEFs) at passages 2–3. For endogenous CIZ1 in UT and C181 cells P = 0.00033 for H3K27me3 P = 0.79, for H2AK119ub1 P = 0.016, performed on present (type 1) categories. Also shown is the effect of 5 μM PR619 on H2AK119ub1 loss, where P = 0.0099 for the no CIZ1 category (type 3). Error bars show SEM. **(C)** Example images of endogenous CIZ1 and histone marks (red) in untransfected (UT) and C181 transfected (green) WT PEF populations. The bar is 10 μm. **(D)** Lentivirus encoding C181 and/or ZSGreen was used to infect three independent populations of WT murine primary embryonic fibroblasts (PEFs) at passage 1–2. Below, expression was verified by western blot of ectopic CIZ1 (exon 17) and beta-actin in whole cell lysates over 3 days, compared to untreated control populations (UT) at days 1 and 3. Below right, live cell images of ZsGreen and brightfield images of PEFs at day 2 after transduction. Bar is 50 μm. **(E)** Comparison of vector-only populations to those transduced with C181 showing the frequency of cells with CIZ1–Xi assemblies (gray), H3K27me3 (red) or H2AK119ub1 (blue). *n* denotes replicate analyses with total nuclei inspected in parentheses. PEF cell populations are in gray. Comparisons are by unpaired *t* test where P < 0.001 in all cases. Error bars show SEM. Below are box and whisker plots showing mean nuclear intensity measures for cells transduced with C181 or vector control, normalized to the mean of vector-only control cells. Source data are available for this figure: SourceData F4.

We postulated that the dispersal of CIZ1–Xi assemblies by DNFs might mimic the effect on Xi chromatin seen in genetically CIZ1 null primary embryonic fibroblasts (PEFs). In these cells, H3K27me3 and H2AK119ub1 are both depleted, and control over PRC target genes, both X-linked genes and elsewhere in the genome, is relaxed (Stewart et al., 2019). In single-cell cycle experiments, in two cell types, C181 caused a marked reduction in H2AK119ub1-enriched Xi’s but did not affect H3K27me3 (Fig. 4, A–C), while in longer-term experiments using lentiviral transduction of C181 (Fig. 4 D) both H3K27me3 and H2AK119ub1 were depleted, whether quantified by enriched Xi frequency or by fluorescence intensity (Fig. 4 E). Survival of H3K27me3 under conditions where H2AK119ub1 is depleted is consistent with replication-linked dilution of H3K27me3 (Coleman and Struhl, 2017; Jadhav et al., 2020; Stewart et al., 2019). Together these data show that DNFs impact histone PTMs.

**Figure 5.**
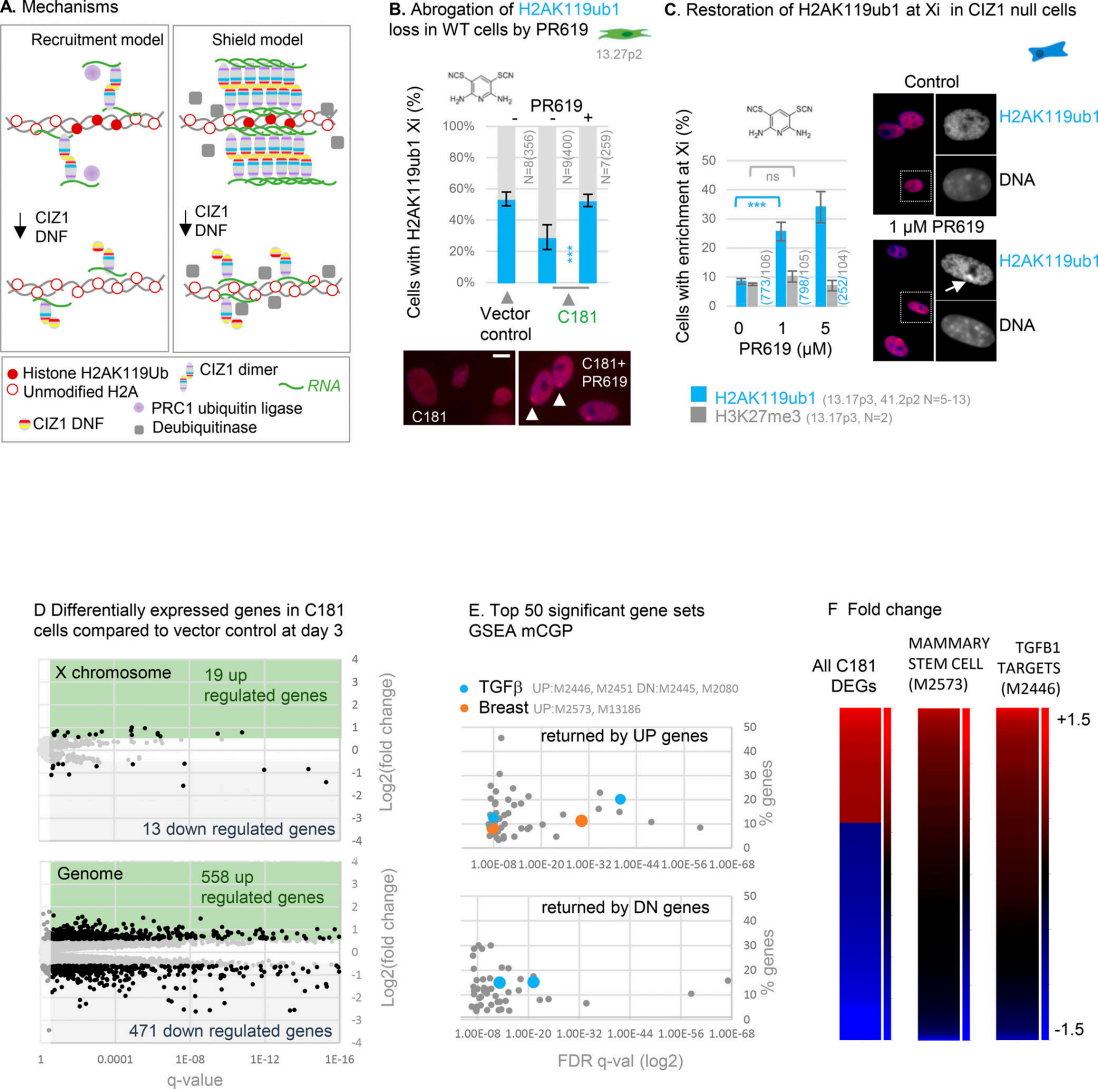
**Effect of CIZ1 anchor domain on gene expression. (A)** Possible mechanisms by which CIZ1 assemblies might influence H2AK119ub1 dynamics on Xi chromatin. Recruitment model: CIZ1–*Xist* assemblies contribute to recruitment or activation of PRC1, supporting H2AK119ub1 deposition. Shield model: Multiple CIZ1 dimers and RNAs coalesce to form a molecular shield at the Xi which blocks access to deubiquitinating enzymes, supporting H2AK119ub1 preservation. **(B)** Frequency of cells with H2AK119ub1 at the Xi in the vector-only population, and cells transduced with C181, without and with the DUB inhibitor PR619 (5 μM). N is replicate analyses with total nuclei inspected in parentheses. Comparisons are by unpaired *t* test. Error bars show SEM. Below are example images taken under standardized conditions showing H2AK119ub1 in red in C181-transduced WT primary embryonic fibroblasts. **(C)** Restoration of H2AK119ub1 enrichment at Xi in CIZ1 null primary embryonic fibroblasts by PR619. In untreated cells ∼10% of cells have H2AK119ub1 enriched Xi’s, which increased to ∼35% within 24 h of treatment, while H3K27me3 remains unchanged. Error bars show SEM. Right, example images of H2AK119ub1 in CIZ1 null primary embryonic fibroblasts. **(D)** Differentially expressed genes in C181 expressing PEFs, compared to vector control, showing log_2_ fold change in FPKM against false-detection rate (FDR) corrected q value, and inclusion threshold of q < 0.05 and absolute log_2_FC > 1. **(E)** Gene set enrichment analysis of all gene sets derived from chemical or genetic perturbation of murine cells (mCGP). Significance indicator is plotted against % genes in overlap for the top 50 sets returned by C181-induced UP genes and C181-induced DN genes. Those linked with TGFβ or breast cells are highlighted in blue and orange respectively, and set identifiers are given in gray. Source data is given in Data S3. **(F)** Heat maps showing C181 DEGs (left, q < 0.05 log_2_FC 1), all genes in mammary stem cell set M2573 (Lim et al., 2010) (middle), and all genes in TGFβ target set M2446 (Plasari et al., 2009) (right), where C181-induced fold change of +1.5 or over is maximally red and less than −1.5 is maximally blue. Gene names and source data are given in Data S3.

Enrichment of H3K27me3 and H2AK119ub1 is sometimes taken as evidence that PRCs were specifically recruited by lncRNAs to the same sites, based in part on extensive but controversial evidence of interaction between PRC subunits and *Xist* (Cech et al., 2024; Guo et al., 2024a, 2024b; Lee and Lee, 2024). However, enrichment of histone PTMs could also arise by a local shift in the balance between addition and removal. In our experiments, disruption of CIZ1–Xi assemblies by DNFs could deplete H2AK119ub1 in Xi chromatin by reducing recruitment of PRC1, or conversely by deprotecting chromatin and allowing access to de-ubiquitinating enzymes. BAP1 is the catalytic subunit of the deubiquitinating enzyme (DUB) that removes H2AK119ub1, acting to restrict its deposition to specific locations (Conway et al., 2021). To begin to distinguish between recruitment and protective functions (Fig. 5 A), we used PR-619, a broad-spectrum reversible inhibitor of DUBs, including the PR-DUB BAP1 (Altun et al., 2011). In one-cell cycle experiments, the immediate (within 24 h) loss of H2AK119ub1 was significantly blocked by PR-619 (Fig. 4 B), and in longer (3 days) transduction experiments, the same trend was observed (Fig. 5 B). Moreover, even in genetically CIZ1 null primary cells, in which H2AK119ub1 is absent from Xi chromatin (Stewart et al., 2019), its enrichment (but not that of H3K27me3) was restored within 24 h of exposure to PR-619 (Fig. 5 C). Thus, loss of CIZ1–Xi assemblies, whether by genetic deletion or dispersal by DNFs, suppresses the accumulation of H2AK119ub1 in Xi chromatin in a manner dependent on DUB activity. We suggest therefore that CIZ1 assemblies perform a shield function that can protect chromatin from enzymatic attack.

### Effect on gene expression

**Figure S3.**
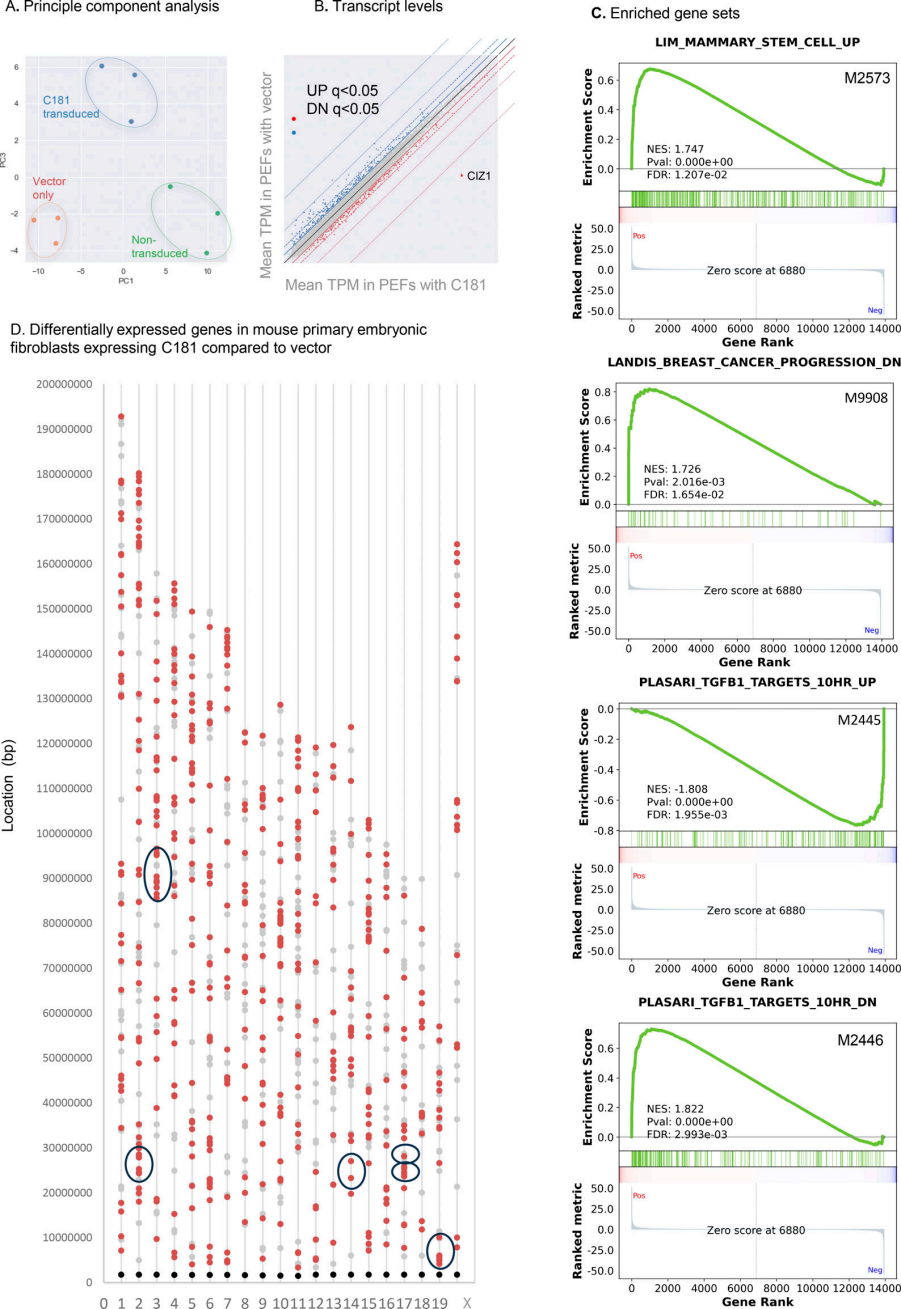
**(Related to** Fig. 5 **). Further analysis of murine genes changed by ectopic expression of C181. (A)** Principle component analysis showing clustering of triplicate PEF-derived transcriptomes for each condition. **(B)** Scatter plot showing mean transcripts per million reads (TPM), colored blue (down, DN) and red (UP) for genes meeting the false-detection rate corrected q values of <0.05 and log_2_ FC 1. **(C)** Example gene set enrichment analysis performed on preranked transcription units derived by comparing expression in C181 transduced cells to the empty vector control, showing highly significant enrichment in two breast stem and cancer-related gene sets, and two controlled by TGFβ. **(D)** Chromosomal locations of C181-driven UP regulated genes (orange) and DN regulated genes (gray, FDR q < 0.05, log_2_FC1), with centromeres shown in black. Circled are regions of synteny to the human cluster regions highlighted in Fig. 7. Human cluster at 1q22 (148–158,000,000) is syntenic with murine chromosome 3 (86,903,019–97,986,449), human cluster at 6p21.31 (25–35,000,000) is syntenic with murine chromosome 17 (17: 27,135,758–31,159,854), human cluster at 9q34 (128–138,000,000) is syntenic with murine chromosome 2 (24,493,799–32,150,031), human cluster at 10q22.2 (70–80,000,000) is syntenic with murine chromosome 14 (20,344,703–25,806,867), human cluster at 11q13.1 (60–70,000,000) is syntenic with murine chromosome 19 (3,309,831–13,840,444), human cluster at 16p13.3 (0–10,000000) is syntenic with murine chromosome 17 (23,765,442–26,506,126).

To confirm that CIZ1 DNFs have the potential to affect gene expression, we analyzed transcriptomes of three independent populations of PEFs, transduced with C181 or empty lentiviral vector for 3 days (Fig. 4 D). To defocus analysis from Xi, we used primary cells isolated from two female and one male murine embryo. This returned expression changes across all chromosomes (Fig. 5 D; and Fig. S3, A and B), including 471 downregulated genes (DN) and 558 upregulated genes (UP, FDR q < 0.05, log_2_FC > 1, Data S3). The 19 UP and 13 DN regulated X-linked genes do not argue for a disproportionate effect on the X chromosome. Gene set enrichment analysis with those that are named coding genes returned highly significant molecular signatures derived by chemical or genetic perturbation in murine cells (GSEA MSig. mCGP), including sets linked with the developmental regulator TGFβ and mammary stem cell phenotype (Fig. S3 C). Looking separately at UP and DN genes, sets related to developing breast tissue and mammary stem cell phenotype are returned primarily by UP genes (Fig. 5 E). Focusing on mammary stem cell phenotype set M2573 (Lim et al., 2010), 25% of all genes in the set are significantly changed by the expression of C181 (FDR q < 0.05, Fig. 5 F), and 75% of those are UP (Data S3). This shows that, similar to germ-line deletion of CIZ1 (Ridings-Figueroa et al., 2017), interference with CIZ1 assemblies in an acute setting can significantly alter gene expression across the genome (Fig. S3 D), including genes linked with cellular plasticity and cancer. Moreover, as in our previous experiments where CIZ1 is reintroduced against a CIZ1 null background (Ridings-Figueroa et al., 2017), the effect is rapid (within days) and coincident with changes to the epigenetic landscape. Together, the data argue for a potent and rapid effect of CIZ1 DNFs that can change established patterns of gene expression.

### Gene expression in human breast cancers

**Figure 6.**
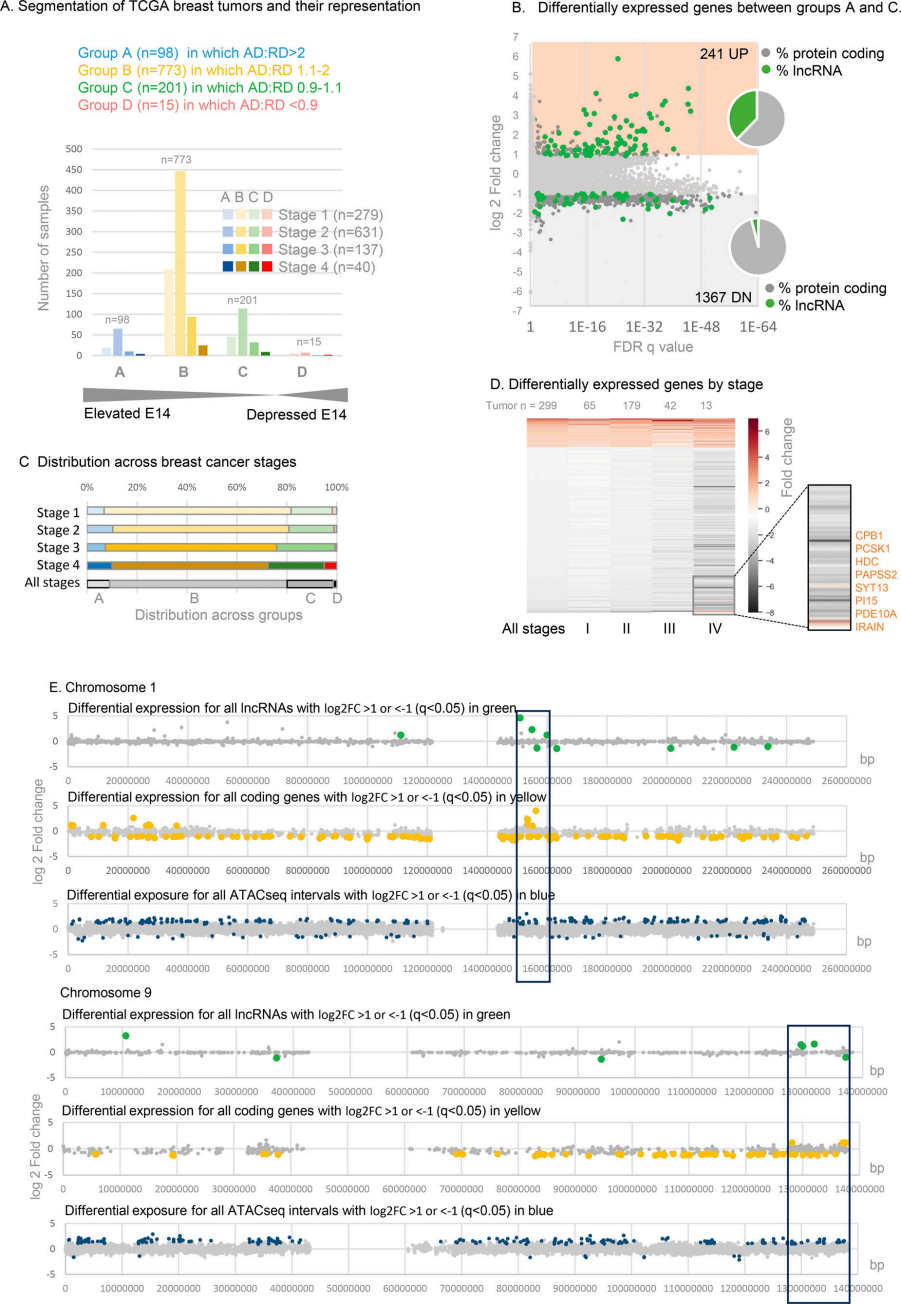
**Gene expression in TCGA breast cancers with elevated CIZ1 anchor domain. (A)** Classification of TCGA breast tumor transcriptomes based on the ratio of CIZ1 AD (exon 14) to RD (exon 5) to create a DNF index comprising groups A–D, shown after segregation by tumor stage. See also Data S4. **(B)** Differentially expressed genes between groups A (greater than twofold elevation of AD) and C (equal ±10%), showing 241 UP (log_2_FC ≥ 1) and 1367 DN (log_2_FC ≤ 1), where q < 0.05. Inset, pie charts show the proportion that are lncRNAs (green) or protein-coding genes (gray). **(C)** TCGA breast cancers are subdivided by stage, showing representation across the DNF index as % (see also Fig. S4). **(D)** Heat map showing all differentially expressed genes derived from comparison of groups A and C, and their representation across stages I–IV. Inset, a highlight of a small subset of mostly enzyme encoding genes whose expression is suppressed in early stages but which switch to UP genes in stage IV disease. **(E)** Example chromosomes 1 and 9 showing, top, differentially expressed lncRNAs (green) returned by comparison of TCGA breast tumors with DNF index A (elevated AD) compared with C (balanced RD and AD). Unaffected genes are shown in gray. Middle, as above for protein-coding genes (yellow). Lower, chromatin accessibility was revealed by ATACseq in eight group A tumors compared with 15 group C tumors, with non-significant intervals in gray and differentially accessible intervals in blue. ATACseq peaks are evident across all chromosomes, and within cluster regions are exclusively UP. The cluster region is marked with a box (10 Mb).

To test whether the disruption of gene expression observed in DNF modeling experiments might be at play in primary human breast cancers, we segmented TCGA breast cancer transcriptomes into four groups A–D (Fig. 6 A and Data S4) based on the extent of elevation of AD over RD. Gene expression in group A tumors in which exon 14:5 ratio is >2, compared with control group C (where RD and AD are within 10% of even), revealed a massive difference in their transcriptomes (1,608 differentially expressed genes [DEGs] FDR q < 0.05, log_2_FC > 1, Fig. 5 B and Data S5).

**Figure S4.**
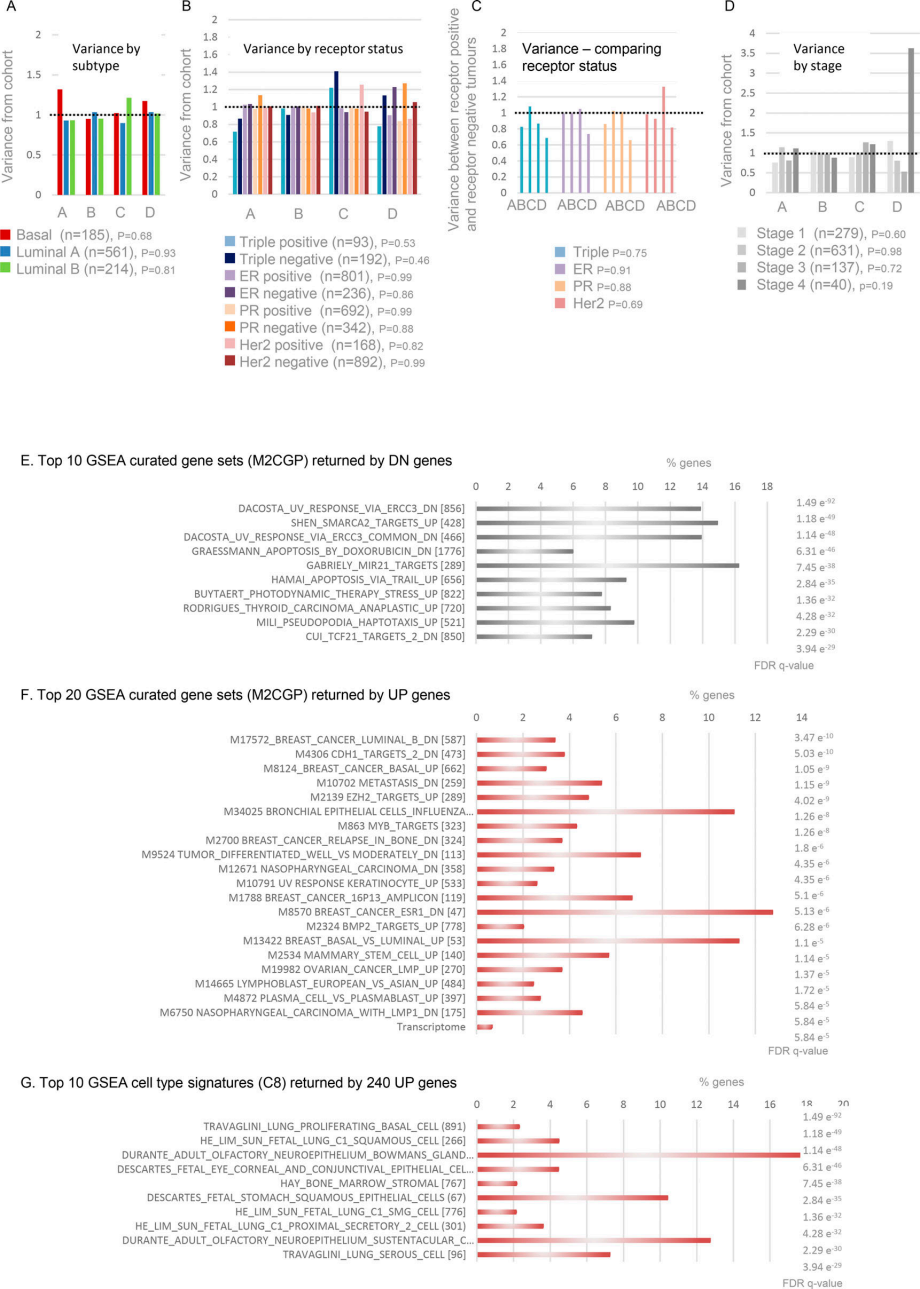
**(Related to** Fig. 6 **). Segmentation of TCGA transcriptomes and GSEA outputs. (A)** TCGA breast cancers by histological subtype (Thennavan et al., 2021), showing representation across the DNF index as variance from the full breast cancer TCGA cohort profile. The number of samples in each subtype is denoted by *n*. Similarity to cohort profile was evaluated by Chi-squared test, where P < 0.05 is considered significant. Dotted line indicates lack of variance. **(B)** As in A, but after classification by receptor status (Thennavan et al., 2021). **(C)** Variance between receptor positive and receptor-negative groups. **(D)** As in A, but after classification by tumor stage. **(E)** Top 10 GSEA curated gene sets (M2 CGP) returned by DN genes, including three related to response to UV and two related to apoptosis. **(F)** Top 20 GSEA curated gene sets (M2 CGP) returned by UP genes, including six breast cancer–related sets, one describing mammary stem cell phenotype and one describing genes normally suppressed by PRC2 catalytic subunit EZH2. **(G)** Top 10 GSEA cell type signatures (C8) returned by UP genes, including primarily fetal cell types but no normal breast tissue signatures.

No significant differences in the proportion of tumors in groups A–D were evident across breast cancer subtypes or ER/PR/HER2 receptor status subsets (Thennavan et al., 2021) (Fig. S4, A–C). Similarly, across tumor stages I, II, and III (all subtypes), group A–D profile is close to the cohort profile (Fig. 6 C and Fig. S4 D), but shifts at stage IV where a greater proportion are group D (AD:RD ratio favors RD). This mirrors a trend observed in stage IV lung, thyroid, and kidney tumors by PCR (Fig. S1 E) in which RD is more likely to exceed AD. In both contexts, however, sample size is too low to draw strong conclusions.

The DEGs between groups A and C behave remarkably similarly across stages I, II, and III (Fig. 6 D), but at stage IV a minority (predominantly enzymes) switch from DN to UP (Fig. 6 D, segment). Overall, the main conclusion to be drawn from this analysis relates to early-stage disease. Not only is C-terminal elevation evident very early in the course of the disease (Fig. 2), its effects are also felt early (stage 1), and those effects persist through to later stages.

### Affected chromosomal domains

**Figure 7.**
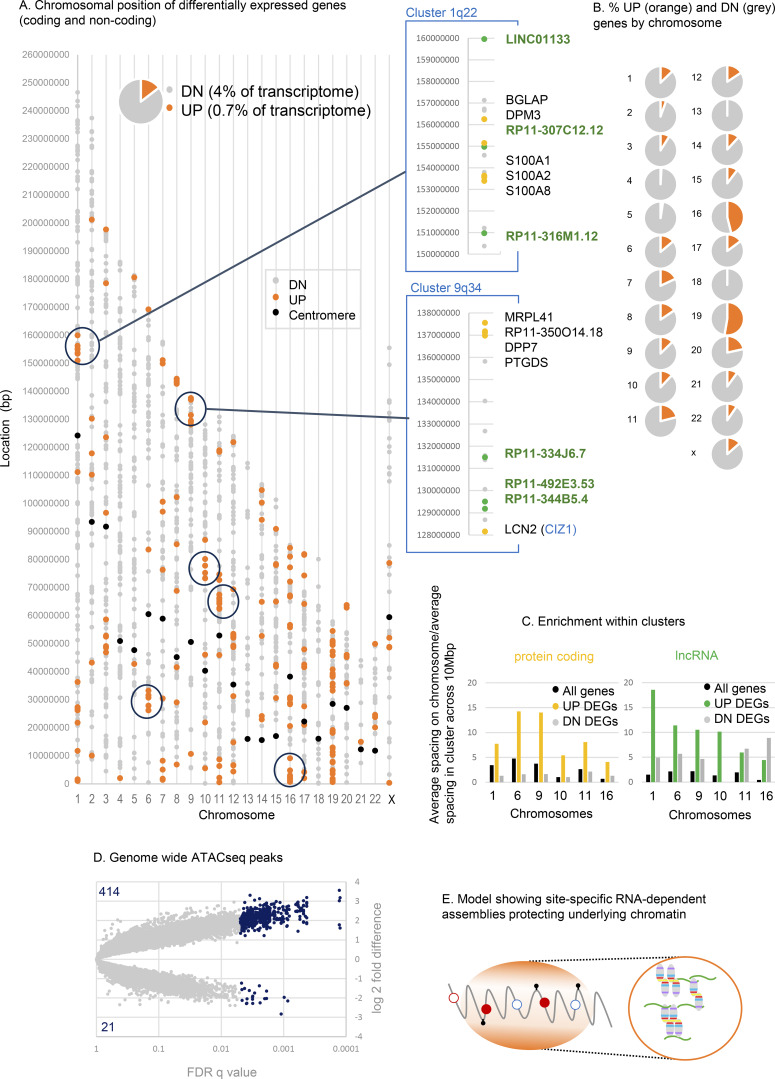
**Affected gene clusters, lncRNA enrichment, and chromatin accessibility. (A)** Chromosomal locations of differentially expressed genes derived from a comparison of TCGA CIZ1 groups A and C (UP orange, DN gray, q < 0.05). Centromere positions in black. Circled clusters are also shown in Fig. S5. Right, two example cluster regions on chromosomes 1 (q22), and 9 (q34). LncRNAs are green, and protein coding genes in yellow. The CIZ1 locus itself is within the circled UP cluster at 9q34. **(B)** Pie charts show the proportion of UP and DN genes by chromosome, highlighting the complete absence of UP genes on 18 and high representation on 19. **(C)** Fold gene enrichment in the indicated 10 Mb clusters compared with chromosomal average for genes (black), and those that are UP (yellow/green) or DN (gray). Protein coding (left) and lncRNAs (right) are shown separately. **(D)** Genome-wide ATACseq differences between group A and group C TCGA breast tumors, showing all intervals in gray and differently accessible intervals in blue (absolute log_2_ FC ≥ 1, q < 0.05). Over 400 sites are significantly more exposed compared with 21 that are less exposed. **(E)** Illustration showing localized CIZ1–RNA assemblies surrounding and modulating access to, underlying chromatin.

**Figure S5.**
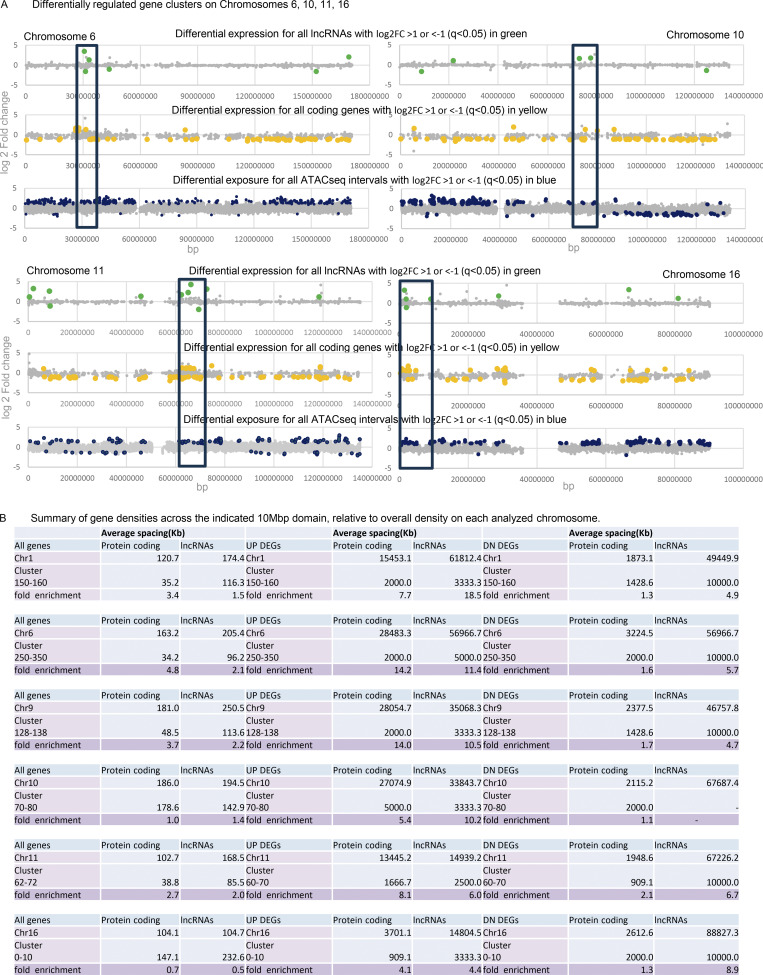
**(Related to** Fig. 6 **and** Fig. 7 **). Focus on UP gene clusters. (A)** Upper, differentially expressed lncRNAs (green) returned by comparison of TCGA breast tumors with DNF index A (elevated AD) compared with C (balanced RD and AD). Unaffected genes are shown in gray. Middle, as above for protein-coding genes (yellow). Lower, chromatin accessibility revealed by ATACseq in 8 group A tumors compared to 15 group C tumors, with non-significant intervals in gray and differentially accessible intervals in blue. ATACseq peaks are evident across all chromosomes, and within cluster regions are exclusively UP. The cluster region is marked with a box (10 Mb), for chromosomes 6,10,11,16. **(B)** Table showing the summary of gene densities across the indicated 10Mbp domains, relative to overall density on each analyzed chromosome. UP clusters encode more genes (coding and non-coding) than the chromosomal averages and are similarly represented in downregulated protein-coding genes but over-represented in upregulated protein coding genes. For lncRNAs, both UP and DN genes are enriched in excess of the gene density.

Of the 1,608 genes that are differentially expressed when CIZ1 AD is overrepresented, 15% are UP and 85% are DN. When analyzed by location, the DN genes are distributed more uniformly than the UP genes, which are clustered (e.g., chromosomes 1 and 9 in Fig. 6 E), are entirely absent from chromosome 18, and are over-represented on gene-dense chromosome 19 (Grimwood et al., 2004) (Fig. 7, A and B). For six gene clusters of 10 Mbp in length (Fig. 7 C, circled in 7 A, Fig. 6 E, and Fig. S5), UP regulated protein-coding genes are 4–14x denser than the chromosomal average but also enriched 2–6x greater than expected for local gene density. In contrast, the frequency of DN-regulated genes reflects local gene density (Fig. S5 B). This spatially concentrated UP-regulation is consistent with a CIZ1-related mechanism that normally represses gene expression across large chromosomal domains.

For the six UP gene clusters, we asked whether syntenic regions were similarly affected in our mouse model. In fact, all were among those regions encoding UP genes in mouse-cultured fibroblasts expressing ectopic AD (Fig. S3 D). Thus, despite differences in species and cell type, and duration and quantity of AD expression, similarities were observed, arguing for a degree of mechanistic conservation.

Notably, among UP genes, 38% encode lncRNAs compared with only 4% of DN genes (Fig. 6 B, listed in Data S5, tab 9). These are concentrated within clusters of UP-regulated protein-coding genes at a density greatly in excess of expected (Fig. 7 C and Fig. S5 B), pointing to a relationship between CIZ1 and lncRNA expression. *Xist* is not among the significantly affected lncRNAs (log_2_FC 0.16, FDR q = 0.0506, Data S5, tab 8).

Interestingly, differentially expressed lncRNAs that are concentrated in cluster regions are both UP- and DN-regulated (Data S5), suggesting functional specialization. By analogy with the CIZ1–*Xist* complexes that form at Xi, we suggest that CIZ1 normally sequesters lncRNA molecules into RNA–protein assemblies (protecting some), which then modulate access to the locus as a whole (repressing others). Excess CIZ1 AD expression would be expected to dissolve the assembly and so release the locus.

Exposure of underlying chromatin by DNF-mediated assembly dissolution alters access by deubiquitylases and might therefore be expected to increase susceptibility to transposases. ATACseq has been performed for a subset of TCGA tumors in group A (*n* = 8) and group C (*n* = 15) to reveal chromatin accessibility across the genome. Using stringent criteria (log_2_FC > 1 or less than −1, FDR q < 0.05), over 400 sites are significantly more exposed in group A than in group C (Fig. 7 D), showing that elevated AD is associated with chromatin accessibility. Exposed sites are located within cluster regions but are also evident in locations that do not host DEGs (Fig. 6 E, Fig. S5 A, and Data S6).

### Affected genes

The many genes (4% of the transcriptome) whose expression is reduced in tumors with elevated AD are distributed across all chromosomes, are not enriched in lncRNAs, and the mean fold change is overall less than UP genes (−1.19 compared to +1.58). Together, this suggests that a different mechanism is at play to that which affects UP genes and, at present, it is difficult to form a strong hypothesis about the process. Alone, they return highly significant enrichment scores for gene sets linked with the cellular response to DNA damaging agents (Fig. S4 E), and when combined with the UP gene set, their over fivefold higher abundance dominates the results.

In contrast, the UP set of 240 spatially regulated genes was highly enriched in breast cancer-related curated gene sets (6 of the top 20 significant overlaps FDR q < 0.05, Fig. S4 F), despite cell-type signatures identifying primarily lung tissue of fetal origin (Fig. S4 G). The UP genes also returned five sets associated with other types of cancer, one describing genes under the regulation of EZH2 (catalytic subunit of PRC2 responsible for H3K27me3) and one describing mammary stem cells. Together these studies support the conclusion that excess expression of CIZ1 AD promotes the expression of genes linked with breast cancer.

Notably, expression of the *CIZ1* gene itself is not returned as UP- or DN-regulated, despite the very different domain expression on which groups C and A were defined. This highlights an important deficiency in the way gene expression analysis is typically carried out, with an amalgamation of all transcripts for a given gene into one indicator. For CIZ1, the common alteration observed here in breast cancers is not evident from overall expression level data, so it has not yet been captured by large-scale transcriptome studies. Furthermore, there are no recurrent polymorphisms in CIZ1 in adult cancers across 46,014 unique samples in COSMIC (Tate et al., 2019), so despite apparently profound effects on breast cancer gene expression, CIZ1 is not yet recognized as a “cancer” gene.

## Discussion

The purpose of heterochromatin formation during development is to protect and reinforce cell fate decisions by restricting access to genes. Thus, potential stabilizers of the chromatin state, whose mis-expression may lead to heterochromatin instability, are important to understand in relation to the degeneration of cellular identity, human disease, and aging. Transcript variants of CIZ1 have been reported in a range of adult and pediatric cancers, as well as in neurological disorders including dystonias (Xiao et al., 2014a) and Alzheimer’s disease (Dahmcke et al., 2008), all of which could be affected via the same primary mechanism of weakened heterochromatin.

Our data suggest that RNA-dependent CIZ1 assemblies, exemplified by the *Xist*- and PLD-dependent CIZ1 SMACs that surround the inactive X chromosome in differentiated cells, normally act as a molecular shield that helps protect heterochromatin from the action of PR-DUBs, and possibly other enzymatic modifiers. “Molecular shield” is one of eight functional classes proposed for phase-separating proteins outlined by PhaSePro (Mészáros et al., 2020). Defined as membraneless organelles that inactivate reactions by sequestering some of the required components while keeping others outside, a CIZ1 shield would sequester chromatin while excluding PR-DUBs. Questions remain about the structure and influence of such a shield and whether some molecules penetrate more freely than others.

### Shield loss

We have exploited the easily visualized Xi-associated CIZ1 assemblies as an indicator of dysfunction in breast cancer cells and as a read out on the solubilizing action of CIZ1 DNFs in a murine model system. Experimentally, the exclusion of the N-terminal RD domain which encodes the two PLDs that confer the ability to coalesce inside the nucleus (Sofi et al., 2022) converts CIZ1 from a SMAC participant into a molecule with the ability to disperse SMACs—a SMAC buster. A shift toward SMAC buster expression is suggested to interfere with normal CIZ1 function in heterochromatin protection and so contribute to epigenetic deprogramming. Importantly, both SMAC buster sequence elevation in breast cancer cells and experimental DNF transgenes alter the transcriptome and, like deletion of CIZ1 (Ridings-Figueroa et al., 2017), effects are felt across the nucleus, with X-linked genes and other chromosomes similarly affected. Thus, while Xi-associated CIZ1 SMACs offer an important model for visual studies, smaller assemblies associated with other chromosomes are likely also disrupted.

The lack of difference in *Xist* expression between breast cancers with and without elevated AD and lack of enrichment of DEGs on the X chromosome has a number of possible explanations: (1) lack of homogeneity in response between active and inactive X chromosomes leading to failure to meet the significance thresholds, (2) cancer-associated changes that are independent of CIZ1 expression, or (3) lack of Xi sensitivity to loss of CIZ1 assemblies possibly buffered by other repressive mechanisms. Notwithstanding this apparent lack of effect on Xi gene expression, we cannot confidently rule out the possibility that changes in autosomal gene expression are not indirect, exerted via subthreshold disruption of X-linked genes (Topa et al., 2024).

### Susceptible loci

Discrete chromosomal domains are susceptible to SMAC busters at the transcript level, suggesting that the protective effect of CIZ1 assemblies is spatially restricted but broad, extending over domains in excess of 10 Mb. Within affected domains, protein-coding genes are over-represented but also disproportionately UP-regulated, implying both domain-wide derepression and concentration of genes within CIZ1-protected clusters.

The behavior of lncRNAs within the same domains is not consistent. While lncRNA genes are also enriched and also more likely to be affected than the chromosomal average, this can be UP or DN. Their heterogenous relationship with AD elevation could reflect more than one mechanism. While UP genes may be subject to the same locus derepression as protein-coding genes, the role of lncRNAs in the formation of spatial compartments in the nucleus (Quinodoz et al., 2021) suggests that others might experience transcript preservation upon incorporation into stable locus-specific RNA-protein SMACs.

Taken together, these data argue that the chromatin deprotection observed in DNF modeling experiments is at play in breast cancers and influences gene expression within specific chromosomal domains, possibly by locally altering the balance between ubiquitination of H2AK119 by PRC1 and its removal by PR-DUBs. Crucially, domain deprotection is evident in early-stage cancers but also persists in later stages, raising the possibility that it is a predisposing influence involved in cancer etiology. At present the question of what drives DNF expression in early-stage breast cancers is unanswered. Lack of mutations in CIZ1 raises the possibility that DNF expression is itself controlled primarily epigenetically and that a normal biological context is yet to be found. If DNFs normally confer fluidity on SMACs, for example, as cells pass through natural transition states, delays imposed by extrinsic conditions might prolong residency and exposure to the destabilizing effect of DNFs.

### Epigenetic origins of cancer

There remain fundamental questions about the relationship between genetic and epigenetic models of cancer and the question of which comes first is likely to have a range of context-specific answers. Mutations in chromatin proteins and their modifiers occur in approximately half of all tumors (You and Jones, 2012), implying that epigenetic instability is a consequence of mutation, yet for some types of tumor no genetic driver mutations are detected (Mack et al., 2014). In fact, it has been shown convincingly that transient depletion of polycomb proteins during Drosophila larval development is sufficient to initiate cancer phenotypes without genetic change (Parreno et al., 2024). Our proposal is that expression of CIZ1 DNFs drives disruption of chromatin state in the early stages of tumor development, possibly before acquisition of driver mutations, and certainly before widespread genetic instability. While the TCGA breast cancer analysis suggests this, direct modeling of the impact of DNFs by the introduction to normal cells shows unequivocally their ability to drive widespread changes in gene expression.

## Materials and methods

### Materials availability and contacts

Further information and requests for resources and reagents should be directed to the lead contacts, G.L. Turvey and D. Coverley.

### Human primary cells

Primary human mammary epithelial cells (HMECs) were cultured at 37°C with 5% CO_2_ in MEBM basal medium (Lonza) supplemented with MEGM SingleQuots (Lonza) on culture dishes coated in collagen (Thistle Scientific) and sampled at passages 1–2. HMECs were acquired with informed consent from three donors by the Breast Cancer Now Tissue bank under NHS ethical approval, and accessed under local approval from the University of York Department of Biology Research Ethics Committee.

### Human cell lines

All cell lines used are of female origin and were authenticated for this study by Eurofins Genomics human cell line authentication service (Eurofins Medigenomix Forensik GmbH), which returned the expected identities with 92–100% confidence in all cases. MCF-10A is a non-tumorigenic epithelial cell line established from the human mammary gland with fibrocystic disease. MCF7 is a poorly aggressive and non-invasive triple receptor–positive human breast cancer cell line established from epithelial cells isolated from a metastatic mammary adenocarcinoma. BT-474 is a human breast cancer cell line established from a malignant ductal carcinoma of the breast that overexpresses human epidermal growth factors receptors 2 (HER-2) and estrogen receptors (ER). SK-BR-3 was established from a malignant adenocarcinoma of the breast that overexpresses HER-2. MDA-MB-231 is a human breast epithelial cancer cell line established from a metastatic poorly differentiated triple-negative mammary adenocarcinoma. Information on receptor status is derived from cell bank annotations and was not independently verified in this study. Cells were cultured in the following media: MCF-10A, MEGM, 5% horse serum, 10 µg/ml hydrocortisone, 20 ng/ml EGF, 500 ng/ml insulin, 100 ng/ml cholera toxin, 1% PSG; MCF7, EMEM, 10% fetal bovine serum (FBS), and 1% penicillin–streptomycin–glutamine (PSG) (Gibco); BT-474 and SK-BR-3, DMEM, 10% FBS, and 1% PSG; MDA-MB-231, DMEM, 5% FBS, and 1% PSG.

### Mouse primary cells

All mouse PEF strains (WT 13.24, 13.31, 13.32, 13.33, 13.27, 45.1fc, and CIZ1 null 13.17, 41.2fa) were derived from day 13 embryos from C57BL/6 mice as previously described (Ridings-Figueroa et al., 2017; Stewart et al., 2019). CIZ1 null mice were generated from C57BL/6 ES clone IST13830B6 (TIGM) harboring a neomycin resistance gene trap inserted downstream of exon 1. The absence of *Ciz1*/CIZ1 in homozygous progeny was confirmed by qPCR, immunofluorescence, and immunoblot. Breeding of mice and all work with animal models was carried out under a UK Home Office license and with the approval of the Animal Welfare and Ethical Review Body at the University of York. PEFs were cultured in 4.5 g/l glucose DMEM containing 10% FBS and 1% PSG up to a maximum of passage 3. After passage 4, these cells are referred to as MEFs and were not used here.

### Mouse cell line

The female D3T3 cell line was cultured as described (Stewart et al., 2019) in DMEM, 10% FBS, 1% PSG (Gibco).

### Site-directed mutagenesis

Mutagenic primers that contain additions, substitutions, or deletions of murine CIZ1 by PCR mutagenesis created for this study are listed in Table 1. All plasmids were sequence verified to confirm mutations (Eurofins TubeSeq Service).

**Table 1.** Resources

Reagent or Resource	Source	Identifier
Antibodies		
N-term CIZ1 Rabbit pAb	Coverley et al. (2005)	1793
C-term CIZ1 Mouse mAb Ex17	FDAB	87 Sofi et al. (2022)
CIZ1 Rabbit pAb Ex17	Novus	NB100-74624
Rabbit H3K27me3	CST	9733S
Rabbit H2AK119ub1	CST	8240
Goat α Rabbit Alexa Fluor 568 (Red)	Invitrogen	A11011
Goat α Mouse Alexa Fluor 488 (Green)	Invitrogen	A11001
Goat α Rabbit Alexa Fluor 488 (Green)	Invitrogen	A11034
Goat α Mouse Alexa Fluor 568 (Red)	Invitrogen	A11031
Histone H3	Abcam	ab1791
β-Actin	Abcam	ab11003
Peroxidase IgG α Rabbit	Jackson	211-032-171
Peroxidase IgG α Mouse	Jackson	155-035-174
Bacterial and virus strains		
*Escherichia coli* DH5α competent cells	Invitrogen	Cat#18265017
*Escherichia coli* Stbl3 competent cells	Invitrogen	Cat#C737303
Chemicals, peptides, and recombinant proteins		
MEBM basal medium	Lonza	Cat#CC-3150
MEGM SingleQuots	Lonza	Cat#CC-4136
HG DMEM	Gibco	Cat#31966-021
Collagen Type I, Rat tail	Thistle Scientific	Cat#50201
X2 Transfection reagent	Mirus	Cat#MIR 6003
PR-619	Bio-Techne	Cat#4482/10
Nocodazole	Sigma-Aldrich	Cat#M1404
Thymidine	Sigma-Aldrich	Cat#T1895
PolyFect transfection reagent	QIAGEN	Cat#301105
CloneAmp HiFi PCR premix	Takara	Cat#639298
QIAprep Spin Miniprep Kit	QIAGEN	Cat#27104
Protease inhibitor cocktail, EDTA-free	BioVision	Cat#K272-1
PreScission protease	GE Healthcare	Cat#27084301
Critical commercial assays		
TissueScan cancer and normal tissue cDNA arrays	Origene	https://www.origene.com/products/tissues/tissuescan
Deposited data		
Murine cell transcriptomes	BMK gene	National Center for Biotechnology Information short Read archive project PRJNA1144651
Experimental models: Cells		
Mouse: Female 3T3 fibroblast cell line	Stewart et al. (2019)	D3T3
Mouse: Primary embryonic fibroblasts (PEFs)	Ridings-Figueroa et al. (2017) and this study	Strain specific identifiers
Human: Primary mammary epithelial cells	Breast Cancer now	HMEC
Human: MCF-10A cell line	ATCC	CRL-10317
Human: MCF7 cell line	ECACC	86012803
Human: BT-474 cell line	TCBSR	BT-474
Human: SK-BR-3 cell line	TCBSR	SK-BR-3
Human: MDA-MB-231 cell line	ATCC	HTB-26
Human: Lenti-X 293T cell line	Takara	Cat#632180
Experimental models: Organisms/strains		
Ciz1 null mice Ciz1Gt(IST13830B6)Tigm	Ridings-Figueroa et al. (2017)	N/A
Oligonucleotides		
qPCR primers		
5′-CAG​GGG​CAT​AAG​GAC​AAA​G-3′	*CIZ1* 13F	P1
5′-TCC​GAG​CCC​TTC​CAC​TCC​TCT​CTG​G-3′	*CIZ1* 15R	P2
5′-CGA​GGG​TGA​TGA​AGA​AGA​GGA-3′	*CIZ1* 14F	P6
5′-CCC​CTG​AGT​TGC​TGT​GAT​A-3′	*CIZ1* 16R	P7
5′-CAC​AAC​TGG​CCA​CTC​CAA​AT-3′	*CIZ1* 5F	P9
5′-CCT​CTA​CCA​CCC​CCA​ATC​G-3′	*CIZ1* 5R	P10
5′-ACA​CAC​CAG​AAG​ACC​AAG​ATT​TAC​C-3′	*CIZ1* 6/7 junction F	P13
5′-TGC​TGG​AGT​GCG​TTT​TTC​CT-3′	*CIZ1* 7R	P14
5′-CAA​CCG​CGA​GAA​GAT​GAC​C-3′		Actin F
5′-TCC​AGG​GCG​ACG​TAG​CAC​A-3′		Actin R
qPCR probes		
5′-CGC​CAG​TCC​TTG​CTG​GGA​CC-3′	*Ciz1* 5	5
5′-CCC​TGC​CCA​GAG​GAC​ATC​GCC-3′	*Ciz1* 7	7
5′-TGG​TCC​TCA​TCT​TGG​CCA​GCA-3′	*Ciz1* 14	14
5′-CAC​GGG​CAC​CAG​GAA​GTC​CA-3′	*Ciz1* 16	16
5′-CCC​TGT​ACG​CCT​CTG​GCC​GT-3′		Actin
Mutagenesis primers		Plasmid generated
FP - 5′-GGA​GAG​ATT​GAG​GTG​AAG​CCG​AGA​GAA​ACA​TCC-3′		GFP-C181 Δ706–713 (CIZ1B)
RP - 5′-GGA​TGT​TTC​TCT​CGG​CTT​CAC​CTC​AAT​CTC​TCC-3′		
FP - 5′-GGA​TTT​CCT​GGT​GCC​AGT​GAT​GAA​AGC​CAA​GAA​CCC​AAG​C		GFP-C181 Δ746–779 (ΔMH3)
RP - 5′-GCT​TGG​GTT​CTT​GGC​TTT​CAT​CAC​TGG​CAC​CAG​GAA​ATC​C-3′		
FP - 5′-CCT​GAC​TGC​ACT​GTT​CTG​ATA​GAA​GCT​TCG​AAT​TCT​GC-3′		GFP-C181 Δ809–845 (Δ37)
RP - 5′-GCA​GAA​TTC​GAA​GCT​TCT​ATC​AGA​ACA​GTG​CAG​TCA​GG-3′		
FP - 5′-GCC​CTC​CTC​CTA​CCA​GCC​ACC​AGC​CCA​GCC-3′		GFP-C181 Δ796–811 (ΔNALTAF)
RP - 5′-GGC​TGG​GCT​GGT​GGC​TGG​TAG​GAG​GAG​GGC-3′		
FP - 5′-ATC​CCC​GAA​TTC​CCG​GGT​CGA​CAA​GGA​GAC​AGG​CAG​CCC		GST-C181 from GST-C275
RP - 5′-GGG​CTG​CCT​GTC​TCC​TTG​TCG​ACC​CGG​GAA​TTC​GGG​GAT-3′		
FP- 5′-GCT​TTG​AGA​GTG​GTC​AAT​TCT​GCA​AGC​AGG​TGA​AGC-3′		GST-C181 Δ689–709 (ΔAcD)
RP- 5′-GCT​TCA​CCT​GCT​TGC​AGA​ATT​GAC​CAC​TCT​CAA​AGC-3′		
FP - 5′-GGA​TTT​CCT​GGT​GCC​AGT​GAT​GAA​AGC​CAA​GAA​CCC​AAG​C-3′		GST- C181 Δ746–779 (ΔMH3)
RP - 5′-GCT​TGG​GTT​CTT​GGC​TTT​CAT​CAC​TGG​CAC​CAG​GAA​ATC​C-3′		
FP - 5′-CCT​GAC​TGC​ACT​GTT​CTG​ATA​GAG​GGA​GC-3′		GST-C181 Δ809–845 (Δ37)
RP - 5′-GCT​CCC​TCT​ATC​AGA​ACA​GTG​CAG​TCA​GG-3′		
5′-AGA​CAG​GCA​GCC​CAG​ATG​AGG-3′		Sequencing primer
Recombinant plasmids		
Murine GFP-C275	Ainscough et al. (2007)	N/A
Murine GFP-C181	This paper	N/A
Murine GFP-C181 CIZ1B	This paper	N/A
Murine GFP-C181 ΔMH3	This paper	N/A
Murine GFP-C181 ΔNALTALF	This paper	N/A
Murine GFP-C181 Δ37	This paper	N/A
Murine GFP-C181 Δ37, ΔMH3	This paper	N/A
Murine GFP-I122	Ainscough et al. (2007)	N/A
psPAX2	Trono Lab unpublished	Addgene plasmid #12260
pMD2.G	Trono Lab unpublished	Addgene plasmid #12259
pLVX-EF1α-IRES-ZsGreen1	Takara	Cat#631982
Software and algorithms		
Gene expression profiling Interactive analysis (GEPIA)	Tang et al. (2017)	http://gepia.cancer-pku.cn/
The cancer genome atlas (TCGA)		https://portal.gdc.cancer.gov/
UCSC genome browser	Kent et al. (2002)	https://genome.ucsc.edu/
Catalogue of somatic Mutations In Cancer (COSMIC)	Tate et al. (2019)	https://www.sanger.ac.uk/tool/cosmic/
Ensembl genome browser	Cunningham et al. (2022)	https://www.ensembl.org/index.html
Functional annotation of the mammalian genome (FANTOM5)	Lizio et al. (2015)	https://fantom.gsc.riken.jp/5/
Cellosaurus	Bairoch (2018)	https://www.cellosaurus.org/
GSEAPY	Fang et al. (2023)	https://gseapy.readthedocs.io/en/latest/introduction.html
FIJI	Schindelin et al. (2012)	https://imagej.net/software/fiji/
IBM SPSS Statistics for Macintosh, version 28.0	IBM	N/A
nfcore/atacseq v2.1.2 workflow	Zenodo	https://doi.org/10.5281/zenodo.8222875
LCsolution software	Shimadzu	N/A
Astra V software	Wyatt	N/A
PXi GenSys software	Syngene	N/A
EVOS Xl digital inverted microscope software	AMG (now Thermo Fisher Scientific)	N/A
Axiovision image acquisition software (SE64 release 4.9.1)	Zeiss	N/A
CLASTR	Bairoch (2018)	https://web.expasy.org/cellosaurus-str-search/
GSEA	Broad Institute	https://www.gsea-msigdb.org/gsea/index.jsp
National center for biotechnology information genes and disease		https://www.ncbi.nlm.nih.gov/books/NBK22266/

### Transient transfection

For analysis in cycling cells, cells were seeded on 13-mm glass coverslips at ∼30% confluency 1 day prior to transfection to produce populations at ∼60% confluency at the time of transfection. Coverslips were transferred to individual wells in a 24-well plate in 500 μl media prior to transfection. For each coverslip 50 μl Opti-MEM Medium (Gibco) was mixed with 1.5 μl X2 Transfection Reagent (Mirus) and 200 ng plasmid DNA (pEGFP-C2 with or without inserts derived from CIZ1), incubated for 30 min, and then applied to cells dropwise. Coverslips were fixed and processed for immunofluorescence typically 24 h later. For contact-inhibited cells, cells were plated across a range of densities by serial dilution 2 days prior to transfection. Coverslips at >90% confluency were selected for transfection and processed as above.

### Cell synchrony

D3T3 cells were arrested in mitosis or S phase using 50 ng/ml nocodazole (Sigma-Aldrich) for 16–24 h or 2.5 mM thymidine (Sigma-Aldrich) for 24 h, respectively. Cells arrested in the M phase were isolated by mitotic shake-off and replated onto glass coverslips for analysis after release. Cells held in the S phase grown on glass coverslips were released by washing twice with PBS and then replacing with fresh media. In transduced cell populations, cells were arrested ∼48 h after transduction for 16–24 h, and then released and analyzed. To facilitate the retention of mitotic cells, coverslips were fixed prior to permeabilization.

### Flow cytometry

Cells were isolated from 9-cm culture plates by trypsinization and resuspended in 100 μl cold PBS to obtain a single cell suspension and then stored at −20°C after the addition of 1.5 ml cold 70% ethanol. For analysis, cells were pelleted and resuspended in PBS (500,000 cells/ml), and 55 μl 10× FACS mix (1 mg/ml propidium iodide, 4% vol/vol Triton X-100, 10xPBS) was added per 500 μl of cell suspension. DNA content was measured using CytoFLEX (Beckman Coulter) at excitation 561 nm/emission 610/20 for detection of the DNA-binding dye propidium iodide (Dean and Jett, 1974). A minimum of 5,000 single cells per sample were recorded for analysis using cell cycle algorithm software FCS Express V7 (Dotmatics).

### Inhibitors

To measure the impact of inhibition of PR-DUBs, 5 µM PR-619 (Bio-Techne) was applied to PEFs 16 h after transduction for 32 h, to collect cells 48 h after transduction. In transient transfection experiments, PR-619 was used at 5 µM for 24 h throughout the transfection window.

### Lentivirus transduction

Bicistronic ZsGreen/C181-bearing virus and ZsGreen alone-bearing virus were produced in the Lenti-X 293T subclone of human embryonic kidney (HEK) cells. 8 × 10^5^ HEK cells were seeded per well in a 6-well plate prior to transfection with plasmids. For transfection of each well, 1 µg transfer vector, 0.75 µg packaging plasmid, and 0.25 µg envelope plasmid diluted in 100 μl optiMEM (Gibco) was mixed with 20 μl of PolyFect transfection reagent (Qiagen) and incubated for 5–10 min at room temperature to allow complex formation. 0.6 ml of cell growth medium was added and gently mixed then transferred to one well. Cells were incubated for 16 h and then media was replaced with fresh growth medium (supplemented with the addition of HEPES to a final concentration of 20 mM). At 48 h after transfection, the supernatant-containing virus was harvested and filtered through a low-protein binding filter (0.45 µm; Sarstedt) to remove HEK debris. Viral supernatant was supplemented with 4 μg/ml polybrene (Sigma-Aldrich) and transferred to recipient PEFs or D3T3 cells. Transduction was monitored by the emergence of cytoplasmic ZsGreen and showed that close to 100% of the cells were transduced after ∼48 h.

### Immunofluorescence

Cells grown on coverslips were washed in cytoskeletal buffer (10 mM PIPES/KOH pH 6.8, 100 mM NaCl, 300 mM sucrose, 1 mM EGTA, and 1 mM MgCl_2_) with 0.1% vol/vol Triton X-100 and fixed in 4% wt/vol paraformaldehyde (PFA). Where indicated, Triton X-100 was left out of CSK (unextracted cells) or an additional 400 mM NaCl was added (high-salt extraction). After fixation, all coverslips were blocked in antibody buffer (AB) (1xPBS, 10 mg/ml BSA, 0.02% wt/vol SDS, and 0.1% vol/vol Triton X-100) for 30 min, incubated with primary antibodies for 1 h at 37°C, washed three times with AB, incubated with secondary antibodies for 1 h at 37°C, washed three times with AB, and mounted on glass slides with Vectashield containing DAPI (Vector Labs). Primary antibodies are detailed in Table 1. Anti-human CIZ1 monoclonal antibody 87 was generated by Fujirebio Diagnostic Antibodies (FDAB). Anti-species antibodies (Thermo Fisher Scientific) labeled with Alexa Fluor 568 (red) or 488 (green) were used for detection in all cases. Fluorescence images were captured using a Zeiss Axiovert 200M fitted with a 63×/1.40 Plan-Apochromat objective and Zeiss filter sets 2, 10, and 15 (G365 FT395 LP420, BP450-490 FT510 BP515-565, and BP546/12 FT580 LP590) using Axiocam 506 mono and Axiovision image acquisition software (SE64 release 4.9.1) through Zeiss Immersol 518F. For each antibody, constant image capture parameters were used to generate image sets within an experiment, on which quantitative analysis was performed, in all cases from unmodified raw images.

### Phenotype scoring in dispersal assays

Cells were classified by eye across replicate experiments and across two to three replicate coverslips per condition within an experiment. Avoidance of bias was achieved by verification by independent workers in all cases and blinded analysis in some cases. In the three-tier scoring system, cells were categorized as either having a compact CIZ1 Xi assembly (type 1) or not (type 3) or were assigned to an intermediate category (type 2) in which CIZ1 assemblies were reduced or diffuse, or made up of locally dispersed particles. Examples are shown. Empty vectors (EV) are used as a negative control and WT-C181 as a positive control in experiments to test the effect of mutants. In transient transfection experiments, untransfected (not green) cells within test populations serve as internal controls on each coverslip.

### Image analysis in dispersal assays

For measurement of the effect of CIZ1 fragments on endogenous CIZ1 or histone PTMs, sets of images including test and control samples were processed in parallel and imaged with identical parameters in one sitting. All intensity measurements were conducted on unedited, unenhanced raw image sets. FIJI identified regions of interest (ROI) within DAPI-stained fields of nuclei using autothresholding with Otsu setting to create a binary mask that defined nuclear perimeters. ROI’s were applied to antibody-detected fluorescence image layers to generate nuclear intensity means, minimum and maxima per ROI, and area of each nucleus. In female nuclei, to obtain a surrogate estimate of Xi-assembly intensity, overall nuclear maxima were used. Where two or more data sets were combined (for example two C181/vector control pairs) from experiments performed on different days or with different PEF populations, data was amalgamated after normalization of values to the average of the control set in each case. For reproduction, images were digitally enhanced to remove background fluorescence or increase brightness using FIJI. Identical manipulations were applied within an experiment, so that for example, the intensity of staining with and without transfection, or before and after extraction, is accurately represented.

### RNA FISH

Female D3T3 cells were transfected with C181 or C275 for 24 h, then processed for detection of *Xist* transcript by RNA-FISH under RNase-free conditions as described previously (Sofi et al., 2022). Briefly, cells grown on coverslips were fixed with 4% PFA on ice for 10 min, rinsed 3X in PBS, and then incubated for 10 min in PBS supplemented with Triton X-100 (0.5%), BSA (0.5%), and vanadyl ribonucleoside complex (VRC, 2 mM). A 11-kb Spe1-Sal1 *Xist* fragment isolated from a full-length mouse *Xist* clone pCMV-*Xist*-PA (26760; Addgene) was fluorescently tagged using BioPrime labeling kit (18094-011; Invitrogen), replacing the biotin with Chromatide 594-5 dUTP (C11400; Invitrogen). Following overnight labeling the reaction was supplemented with Cot1 and salmon sperm DNA to compete for repetitive elements. The mix was repeat-precipitated twice and then resuspended in 80 µl hybridization buffer comprising 50% formamide in 2X SSC with BSA (2 mg/ml), dextran sulfate (10%), and VRC (10 mM). Prior to use, the probe (10 µl/coverslip) was denatured at 74°C for 10 min and then annealed at 37°C for 20 min. Coverslips were dehydrated through an ethanol series and air-dried. The probe was spotted onto a clean RNase-free slide, overlaid with the coverslip, and then sealed with rubber cement and incubated overnight at 37°C in the dark. The coverslips were then carefully removed in 4X SSC, washed three times in 2X SSC with 50% formamide at 39°C, three times in 2X SSC at 39°C, once in 1X SSC at room temperature, and then once in 4X SSC at room temperature. All washes were for 5 min. Coverslips were briefly dipped in water and mounted in vectorshield with dapi.

### Quantitative RT-PCR

To quantify the relative expression of CIZ1 amplicons in a wide range of primary tumors, TissueScan Tumour cDNA arrays from OriGene Technologies, Inc. containing 2–3 ng of cDNA were analyzed by qPCR. The RNA was collected under IRB-approved protocols, and array details with tumor classification are given in Data S2. Tumor classifications and abstracted pathology reports are given at: http://www.origene.com/qPCR/Tissue-qPCR-Arrays.aspx. cDNA was normalized using β-actin by the supplier, and we used our own amplification of β-actin where indicated. In most cases, results for CIZ1 amplicon expression were expressed relative to one another, rather than to another gene, and normalized to control samples so that change in ratio across cancer samples is apparent. Reactions were carried out in 25 μl volumes with 12.5 μl Taqman master mix (Applied Biosystems), 1 μl of each 10 µM primer, and 1 μl 10 µM probe. Primers (Sigma-Aldrich) and probes (MWG) are specified in Table 1. Primers 9 and 10 were combined with a probe in exon 5 to generate detection tool set DT5, primers 13 and 14 with probe 7 (DT7), primers 1 and 2 with probe 14 (DT14), and primers 6 and 7 with probe 16 (DT16). Primer efficiencies were >90% in all cases, and the relative amplification efficiencies of RD and AD tools were routinely checked using a plasmid template with coupled and equal levels of RD and AD. Data were generated using an ABI 7000, SDS v1.2. (Applied Biosystems) using 50°C (2 min), 95°C (10 min), then 50 cycles of 95°C (15 s), 60°C (1 min). Relative expression was calculated using the comparative Ct method using the formula, 2^−ΔΔCt^ (Livak and Schmittgen, 2001), and results were expressed relative to the mean of normal cells or tissue in each array, or to the lowest stage tumor in the array, as indicated.

### Mouse transcriptomics

Primary murine embryonic fibroblasts 13.31, 13.32 (WT female), and 13.33 (WT male) were transduced with virus bearing either the empty pLVX-EF1a-IRES-ZsGreen1 vector (Takara) or the same plasmid expressing the coding sequence of C181 as described in lentivirus transduction. After 72 h, RNA was extracted with TRIzol (15596-026; Ambion) following manufacturer’s instructions and RNA pellets were resuspended in nuclease-free water. Isolated RNA was treated with DNase (04716728001; Roche) before quality analysis by agarose gel, NanoDrop spectrophotometer, and Agilent 2100 Bioanalyzer. Library preparation and sequencing were undertaken by Biomarker Technologies (BMKGene), using the NEBNext UltraTM RNA Library Prep Kit for Illumina (NEB), with enrichment for mRNA using oligo(dT)-magnetic beads, followed by random fragmentation of enriched mRNA in fragmentation buffer. cDNA was synthesized using random primers followed by purification with AMPure XP beads, end repair, dA-tailing, adaptor ligation, PCR enrichment, and further AMPure XP purification to select fragments within the size range of 300–400 bp. Library quality was assessed using the Agilent 2100 Bioanalyzer system and sequenced using an Illumina platform using paired-end sequencing to generate at least 15.31 Gb clean data per sample, with a minimum 93.16% of clean data, and a quality score of Q30. Low-quality sequence reads and adaptor sequences were removed and the resulting high-quality reads were aligned to version GRCm38 of the mouse genome using HISAT2 (Kim et al., 2015). Transcriptomes were assembled and gene expression was quantified using StringTie (Pertea et al., 2015) against the Ensemble release 79 annotations. Differential gene expression analysis was performed by DESeq2 (Subramanian et al., 2005). Plots were generated from the average of transcripts per million plus one (TPM+1) values for each treatment condition to exclude very lowly expressed transcripts using Spyder (v.5.3.3) accessed by Anaconda Distribution (v.2.3.2). Scatter plots were generated using the pandas, numpy, and matplotlib modules. Volcano plots were generated using the pandas and bioinfokit modules. Principle component analysis (PCA) plots were generated using the pandas, sklearn, seaborn, and matplotlib modules. Gene set enrichment analysis was performed using the GSEAPY module, with genes preranked based on the generation of a π value (Xiao et al., 2014b) as calculated by multiplication of the log_2_FC of the average TPM by the −log_10_ (q value), and also separately for UP and DN DEGs.

### Patient and cell line bioinformatics

Aligned RNA sequencing data for 1,095 primary breast cancer samples from The Cancer Genome Atlas were accessed under dbGaP project 25297. Secondary metastatic breast samples were excluded from the analysis. Data were downloaded using the Genomic Data Commons command line client v1.5.0. FASTQ files were regenerated from sample BAM files using samtools v1.10 (Li et al., 2009) to exclude secondary and supplementary alignments, and then BEDTools v2.27.1 bamToFastq (Quinlan and Hall, 2010). No additional quality control steps were performed on the extracted read files. Reads were aligned to the GRCh38 Gencode primary assembly and to individual CIZ1 transcripts (from Gencode v38 and [Veiga et al., 2022]) using HISAT2 v2.2.0 (Kim et al., 2015). Reads were also pseudoaligned to the Gencode v38 full annotation transcriptome file with kallisto v0.46.0 (Bray et al., 2016) and quantified and aggregated to gene-level transcripts per million (TPM) expression values using tximport v1.24.0 (Soneson et al., 2015). The same expression analysis pipeline was also completed on publicly available RNA sequencing data from four breast cancer cell lines: MCF7 (SRR8615758), BT-474 (SRR8616195), SK-BR-3 (SRR8615677), and MDA-MB-231 SRR8615767), and the breast epithelium transformed cell lines MCF-10A (SRR12877369).

### Analysis of domain imbalance

CIZ1 encodes 16 translated exons (2–17), plus at least three alternative untranslated exons 1 s which were excluded from the present analysis. CIZ1 exon transcript read coverage in TCGA breast tumors was generated by alignments to both transcript and full genome and inspected manually for novel, well-supported splice junctions in IGV Desktop for Windows v2.8.2 (Soneson et al., 2015). Outputs were normalized to the canonical (ENST00000372938.10) exon 7 coverage and stratified by the tumor stage. To develop an index of the degree of CIZ1 AD and RD domain imbalance, the ratio of exon 14 to exon 5 was calculated for individual tumors after mapping to the CIZ1 transcript. The 10 stage II patients with the most imbalanced AD:RD were used to rule out broader imbalance in 3′ coverage within the libraries. These were TCGA-E9-A54Y, TCGA-LL-A6FR, TCGA-E9-A3X8, TCGA-AQ-A54O, TCGA-AQ-A54N, TCGA-WT-AB41, TCGA-A2-A3XV, TCGA-LL-A5YL, TCGA-GM-A2DB, and TCGA-AO-A03N, which were similar across *ESR1* and *TP53* exons. To compare gene expression in TCGA tumors with and without domain imbalance, a subgroup A (*n* = 98) in which AD (exon 14):RD (exon5) normalized TPM ratio exceeds 2, and a subgroup C (*n* = 201) in which the ratio is within 0.9–1.1 (10% variance) were identified (Data S4). Intermediate group B (ratio 1.1–1.99, *n* = 773) and group D (<0.9, *n* = 15) are also shown. DEGs (absolute log_2_ fold change >1, FDR q-value 0.05) between groups A and C reveal those whose expression correlates with elevated AD. The representation of groups A–D across breast cancer subtypes was based on histological classifications (Thennavan et al., 2021) and on tumor stage classifications associated with accessed transcriptomes (Thennavan et al., 2021) (listed in Data S4) and compared with the cohort as a whole.

### ATACseq analysis

Aligned ATAC sequencing data for all available sub group A (*n* = 8) and subgroup C (*n* = 15) primary breast cancer samples from The Cancer Genome Atlas were accessed under dbGaP project 25297. BAM files were filtered to remove unmapped reads and sorted by read name using SAMtools before conversion to FASTQ format using BEDTools bamToFastq. FASTQ files were processed and analyzed using the nfcore/atacseq v2.1.2 workflow using default parameters. DESeq2 was used to identify differentially accessible regions based on read counts within consensus peaks, stringently filtered for absolute log_2_ fold change >1, FDR q-value 0.05.

### Positional analysis

Chromosome maps were generated by segregating DEGs by UP or DN and then by chromosome, and plotting by the start position of each mapped gene in Excel. Centromere positions were extracted from mouse genome build GRCm39/mm39 and human GRCh38.p14 using the USCC genome browser. The plotted murine gene cohort includes 1,029 C181 DEGs (FDR q < 0.05, log_2_FC > 1 or less than −1), and human gene cohort is 1,126 DEGs related to twofold elevated AD in TCGA patients (FDR q < 0.05, log_2_FC > 1 or less than −1). Average UP and DN gene density was calculated based on sequenced human chromosome lengths given in NCBI Genes and Disease, and locations enriched for UP genes selected based on increased density across 10 Mb domains. For the six human clusters analyzed, UP DEG enrichment exceeded gene enrichment by 2–12-fold. Syntenic regions in the murine genome for human UP gene clusters were identified using the Ensemble Chromosome view synteny tab.

### Quantification and statistical analysis

For analysis of CIZ1 SMAC frequencies, a variable number (>3) of technical replicates and independent counts (*N* value) were conducted per experiment as indicated, allowing the generation of ±SEM. The number of cells scored is stated individually in each experiment (*n* value). Wherever possible, at least two independently isolated PEF lines were used across the experimentation relating to each question. Statistical analysis was carried out using SPSS release 2021 ver.28.0 for Mac (IBM) or Microsoft Excel for Mac ver.16.73.3 Parametric or non-parametric tests were utilized where appropriate; for comparison between two data sets, a two-sample unpaired *t* test or a Mann–Whitney U test was utilized, and for comparison between three or more data sets a one-way ANOVA was followed by an appropriate post-hoc test. Statistical tests used in each analysis are stated in the figure legend with *P* values, where asterisks indicate *P < 0.05, **P < 0.01, and ***P < 0.001. For qRTPCR data, the Pearson correlation coefficient was used to compare CIZ1 RD and AD amplicon expression, and linear regression trendlines were applied using Excel. Graphs were generated using Excel.

### Online supplemental material

Fig. S1 shows CIZ1 domains, transcript levels, and domain expression in common solid tumors. Fig. S2 shows cell cycle analysis and anchor domain mutagenesis. Fig. S3 shows further analysis of murine genes changed by ectopic expression of C181. Fig. S4 shows the segmentation of TCGA transcriptomes and GSEA outputs. Fig. S5 shows a focus on UP gene clusters. Data S1 shows CIZ1 exon expression (cell lines and tumor summary). Data S2 shows data and primary tumor designations for QRTPCR array analysis. Data S3 shows the DNF effect on murine transcriptomics, GSEA analysis, and heat maps. Data S4 shows TCGA BRCA transcriptomics, segmentation, and clinical metadata. Data S5 shows gene expression in BRCA group A compared with C. Data S6 shows ATACseq in group A compared with C.

## Supplementary Material

Data S1 shows CIZ1 exon expression (cell lines and tumor summary).

Data S2 shows data and primary tumor designations for QRTPCR array analysis.

Data S3 shows the DNF effect on murine transcriptomics, GSEA analysis, and heat maps.

Data S4 shows TCGA BRCA transcriptomics, segmentation, and clinical metadata.

Data S5 shows gene expression in BRCA group A compared with C.

Data S6 shows ATACseq in group A compared with C.

SourceData F4 is the source file for Fig. 4.

## Data Availability

The data underlying Fig. 2, A–C, Fig. 5, D–F, Fig.6, and Fig.7 are available in the published article or its online supplemental material. The source data underlying Fig. 2 D, Fig. 6, and Fig. 7 are openly available in The Cancer Genome Atlas (TCGA) at https://www.cancer.gov/ccg/research/genome-sequencing/tcga. All other data are available from the corresponding author upon reasonable request.

## References

[bib1] Ainscough, J.F., F.A. Rahman, H. Sercombe, A. Sedo, B. Gerlach, and D. Coverley. 2007. C-terminal domains deliver the DNA replication factor Ciz1 to the nuclear matrix. J. Cell Sci. 120:115–124. 10.1242/jcs.03327 17182902

[bib2] Altun, M., H.B. Kramer, L.I. Willems, J.L. McDermott, C.A. Leach, S.J. Goldenberg, K.G. Kumar, R. Konietzny, R. Fischer, E. Kogan, . 2011. Activity-based chemical proteomics accelerates inhibitor development for deubiquitylating enzymes. Chem. Biol. 18:1401–1412. 10.1016/j.chembiol.2011.08.018 22118674

[bib3] Bairoch, A. 2018. The cellosaurus, a cell-line knowledge resource. J. Biomol. Tech. 29:25–38. 10.7171/jbt.18-2902-002 29805321 PMC5945021

[bib4] Batra, R.N., A. Lifshitz, A.T. Vidakovic, S.F. Chin, A. Sati-Batra, S.J. Sammut, E. Provenzano, H.R. Ali, A. Dariush, A. Bruna, . 2021. DNA methylation landscapes of 1538 breast cancers reveal a replication-linked clock, epigenomic instability and cis-regulation. Nat. Commun. 12:5406. 10.1038/s41467-021-25661-w 34518533 PMC8437946

[bib5] Bray, N.L., H. Pimentel, P. Melsted, and L. Pachter. 2016. Near-optimal probabilistic RNA-seq quantification. Nat. Biotechnol. 34:525–527. 10.1038/nbt.3519 27043002

[bib6] Brockdorff, N., A. Ashworth, G.F. Kay, V.M. McCabe, D.P. Norris, P.J. Cooper, S. Swift, and S. Rastan. 1992. The product of the mouse Xist gene is a 15 kb inactive X-specific transcript containing no conserved ORF and located in the nucleus. Cell. 71:515–526. 10.1016/0092-8674(92)90519-I 1423610

[bib7] Brown, C.J., B.D. Hendrich, J.L. Rupert, R.G. Lafrenière, Y. Xing, J. Lawrence, and H.F. Willard. 1992. The human XIST gene: Analysis of a 17 kb inactive X-specific RNA that contains conserved repeats and is highly localized within the nucleus. Cell. 71:527–542. 10.1016/0092-8674(92)90520-M 1423611

[bib8] Cech, T.R., C. Davidovich, and R.G. Jenner. 2024. PRC2-RNA interactions: Viewpoint from Tom Cech, Chen Davidovich, and Richard Jenner. Mol. Cell. 84:3593–3595. 10.1016/j.molcel.2024.09.010 39366348

[bib9] Chaligné, R., T. Popova, M.A. Mendoza-Parra, M.A. Saleem, D. Gentien, K. Ban, T. Piolot, O. Leroy, O. Mariani, H. Gronemeyer, . 2015. The inactive X chromosome is epigenetically unstable and transcriptionally labile in breast cancer. Genome Res. 25:488–503. 10.1101/gr.185926.114 25653311 PMC4381521

[bib10] Coleman, R.T., and G. Struhl. 2017. Causal role for inheritance of H3K27me3 in maintaining the OFF state of a Drosophila HOX gene. Science. 356:eaai8236. 10.1126/science.aai8236 28302795 PMC5595140

[bib11] Conway, E., F. Rossi, D. Fernandez-Perez, E. Ponzo, K.J. Ferrari, M. Zanotti, D. Manganaro, S. Rodighiero, S. Tamburri, and D. Pasini. 2021. BAP1 enhances Polycomb repression by counteracting widespread H2AK119ub1 deposition and chromatin condensation. Mol. Cell. 81:3526–3541.e8. 10.1016/j.molcel.2021.06.020 34186021 PMC8428331

[bib12] Coverley, D., J. Marr, and J. Ainscough. 2005. Ciz1 promotes mammalian DNA replication. J. Cell Sci. 118:101–112. 10.1242/jcs.01599 15585571

[bib13] Cunningham, F., J.E. Allen, J. Allen, J. Alvarez-Jarreta, M.R. Amode, I.M. Armean, O. Austine-Orimoloye, A.G. Azov, I. Barnes, R. Bennett, . 2022. Ensembl 2022. Nucleic Acids Res. 50:D988–D995. 10.1093/nar/gkab1049 34791404 PMC8728283

[bib14] Dahmcke, C.M., S. Büchmann-Møller, N.A. Jensen, and C. Mitchelmore. 2008. Altered splicing in exon 8 of the DNA replication factor CIZ1 affects subnuclear distribution and is associated with Alzheimer’s disease. Mol. Cell. Neurosci. 38:589–594. 10.1016/j.mcn.2008.05.007 18583151

[bib15] Dean, P.N., and J.H. Jett. 1974. Mathematical analysis of DNA distributions derived from flow microfluorometry. J. Cell Biol. 60:523–527. 10.1083/jcb.60.2.523 4855906 PMC2109170

[bib16] Dixon-McDougall, T., and C.J. Brown. 2022. Multiple distinct domains of human XIST are required to coordinate gene silencing and subsequent heterochromatin formation. Epigenetics Chromatin. 15:6. 10.1186/s13072-022-00438-7 35120578 PMC8815261

[bib17] Fang, Z., X. Liu, and G. Peltz. 2023. GSEApy: A comprehensive package for performing gene set enrichment analysis in Python. Bioinformatics. 39:btac757. 10.1093/bioinformatics/btac757 36426870 PMC9805564

[bib18] Flavahan, W.A., E. Gaskell, and B.E. Bernstein. 2017. Epigenetic plasticity and the hallmarks of cancer. Science. 357:eaal2380. 10.1126/science.aal2380 28729483 PMC5940341

[bib19] Grimwood, J., L.A. Gordon, A. Olsen, A. Terry, J. Schmutz, J. Lamerdin, U. Hellsten, D. Goodstein, O. Couronne, M. Tran-Gyamfi, . 2004. The DNA sequence and biology of human chromosome 19. Nature. 428:529–535. 10.1038/nature02399 15057824

[bib20] Guo, J.K., M.R. Blanco, and M. Guttman. 2024a. PRC2-RNA interactions: Viewpoint from Jimmy K. Guo, Mario R. Blanco, and Mitchell Guttman. Mol. Cell. 84:3578–3585. 10.1016/j.molcel.2024.09.007 39366346

[bib21] Guo, J.K., M.R. Blanco, W.G. Walkup IV, G. Bonesteele, C.R. Urbinati, A.K. Banerjee, A. Chow, O. Ettlin, M. Strehle, P. Peyda, . 2024b. Denaturing purifications demonstrate that PRC2 and other widely reported chromatin proteins do not appear to bind directly to RNA in vivo. Mol. Cell. 84:1271–1289.e12. 10.1016/j.molcel.2024.01.026 38387462 PMC10997485

[bib22] Hall, L.L., M. Byron, G. Pageau, and J.B. Lawrence. 2009. AURKB-mediated effects on chromatin regulate binding versus release of XIST RNA to the inactive chromosome. J. Cell Biol. 186:491–507. 10.1083/jcb.200811143 19704020 PMC2733744

[bib23] Hanahan, D. 2022. Hallmarks of cancer: New dimensions. Cancer Discov. 12:31–46. 10.1158/2159-8290.CD-21-1059 35022204

[bib24] Higgins, G., K.M. Roper, I.J. Watson, F.H. Blackhall, W.N. Rom, H.I. Pass, J.F. Ainscough, and D. Coverley. 2012. Variant Ciz1 is a circulating biomarker for early-stage lung cancer. Proc. Natl. Acad. Sci. USA. 109:E3128–E3135. 10.1073/pnas.1210107109 23074256 PMC3494940

[bib25] Jadhav, U., E. Manieri, K. Nalapareddy, S. Madha, S. Chakrabarti, K. Wucherpfennig, M. Barefoot, and R.A. Shivdasani. 2020. Replicational dilution of H3K27me3 in mammalian cells and the role of poised promoters. Mol. Cell. 78:141–151.e5. 10.1016/j.molcel.2020.01.017 32027840 PMC7376365

[bib26] Kent, W.J., C.W. Sugnet, T.S. Furey, K.M. Roskin, T.H. Pringle, A.M. Zahler, and D. Haussler. 2002. The human genome browser at UCSC. Genome Res. 12:996–1006. 10.1101/gr.229102 12045153 PMC186604

[bib27] Kim, D., B. Langmead, and S.L. Salzberg. 2015. HISAT: A fast spliced aligner with low memory requirements. Nat. Methods. 12:357–360. 10.1038/nmeth.3317 25751142 PMC4655817

[bib28] Lee, Y., and J.T. Lee. 2024. PRC2-RNA interactions: Viewpoint from YongWoo lee and Jeannie T. Lee. Mol. Cell. 84:3586–3592. 10.1016/j.molcel.2024.09.006 39366347

[bib29] Li, H., B. Handsaker, A. Wysoker, T. Fennell, J. Ruan, N. Homer, G. Marth, G. Abecasis, R. Durbin, and 1000 Genome Project Data Processing Subgroup. 2009. The sequence alignment/map format and SAMtools. Bioinformatics. 25:2078–2079. 10.1093/bioinformatics/btp352 19505943 PMC2723002

[bib30] Lim, E., D. Wu, B. Pal, T. Bouras, M.L. Asselin-Labat, F. Vaillant, H. Yagita, G.J. Lindeman, G.K. Smyth, and J.E. Visvader. 2010. Transcriptome analyses of mouse and human mammary cell subpopulations reveal multiple conserved genes and pathways. Breast Cancer Res. 12:R21. 10.1186/bcr2560 20346151 PMC2879567

[bib31] Livak, K.J., and T.D. Schmittgen. 2001. Analysis of relative gene expression data using real-time quantitative PCR and the 2(-Delta Delta C(T)) Method. Methods. 25:402–408. 10.1006/meth.2001.1262 11846609

[bib32] Lizio, M., J. Harshbarger, H. Shimoji, J. Severin, T. Kasukawa, S. Sahin, I. Abugessaisa, S. Fukuda, F. Hori, S. Ishikawa-Kato, . 2015. Gateways to the FANTOM5 promoter level mammalian expression atlas. Genome Biol. 16:22. 10.1186/s13059-014-0560-6 25723102 PMC4310165

[bib33] Locke, W.J., and S.J. Clark. 2012. Epigenome remodelling in breast cancer: Insights from an early in vitro model of carcinogenesis. Breast Cancer Res. 14:215. 10.1186/bcr3237 23168266 PMC4053120

[bib34] Locke, W.J., E. Zotenko, C. Stirzaker, M.D. Robinson, R.A. Hinshelwood, A. Stone, R.R. Reddel, L.I. Huschtscha, and S.J. Clark. 2015. Coordinated epigenetic remodelling of transcriptional networks occurs during early breast carcinogenesis. Clin. Epigenetics. 7:52. 10.1186/s13148-015-0086-0 25960784 PMC4424562

[bib35] Mack, S.C., H. Witt, R.M. Piro, L. Gu, S. Zuyderduyn, A.M. Stütz, X. Wang, M. Gallo, L. Garzia, K. Zayne, . 2014. Epigenomic alterations define lethal CIMP-positive ependymomas of infancy. Nature. 506:445–450. 10.1038/nature13108 24553142 PMC4174313

[bib36] Markaki, Y., J. Gan Chong, Y. Wang, E.C. Jacobson, C. Luong, S.Y.X. Tan, J.W. Jachowicz, M. Strehle, D. Maestrini, A.K. Banerjee, . 2021. Xist nucleates local protein gradients to propagate silencing across the X chromosome. Cell. 184:6174–6192.e32. 10.1016/j.cell.2021.10.022 34813726 PMC8671326

[bib37] Mészáros, B., G. Erdős, B. Szabó, É. Schád, Á. Tantos, R. Abukhairan, T. Horváth, N. Murvai, O.P. Kovács, M. Kovács, . 2020. PhaSePro: The database of proteins driving liquid-liquid phase separation. Nucleic Acids Res. 48:D360–D367. 31612960 10.1093/nar/gkz848 PMC7145634

[bib38] Moore, K.L., and M.L. Barr. 1957. The sex chromatin in human malignant tissues. Br. J. Cancer. 11:384–390. 10.1038/bjc.1957.45 13499787 PMC2073881

[bib39] Parreno, V., V. Loubiere, B. Schuettengruber, L. Fritsch, C.C. Rawal, M. Erokhin, B. Győrffy, D. Normanno, M. Di Stefano, J. Moreaux, . 2024. Transient loss of Polycomb components induces an epigenetic cancer fate. Nature. 629:688–696. 10.1038/s41586-024-07328-w 38658752 PMC11096130

[bib40] Pertea, M., G.M. Pertea, C.M. Antonescu, T.C. Chang, J.T. Mendell, and S.L. Salzberg. 2015. StringTie enables improved reconstruction of a transcriptome from RNA-seq reads. Nat. Biotechnol. 33:290–295. 10.1038/nbt.3122 25690850 PMC4643835

[bib41] Plasari, G., A. Calabrese, Y. Dusserre, R.M. Gronostajski, A. McNair, L. Michalik, and N. Mermod. 2009. Nuclear factor I-C links platelet-derived growth factor and transforming growth factor beta1 signaling to skin wound healing progression. Mol. Cell. Biol. 29:6006–6017. 10.1128/MCB.01921-08 19752192 PMC2772573

[bib42] Quinlan, A.R., and I.M. Hall. 2010. BEDTools: A flexible suite of utilities for comparing genomic features. Bioinformatics. 26:841–842. 10.1093/bioinformatics/btq033 20110278 PMC2832824

[bib43] Quinodoz, S.A., J.W. Jachowicz, P. Bhat, N. Ollikainen, A.K. Banerjee, I.N. Goronzy, M.R. Blanco, P. Chovanec, A. Chow, Y. Markaki, . 2021. RNA promotes the formation of spatial compartments in the nucleus. Cell. 184:5775–5790.e30. 10.1016/j.cell.2021.10.014 34739832 PMC9115877

[bib44] Rahman, F., J.F.-X. Ainscough, N. Copeland, and D. Coverley. 2007. Cancer-associated missplicing of exon 4 influences the subnuclear distribution of the DNA replication factor CIZ1. Hum. Mutat. 28:993–1004. 10.1002/humu.20550 17508423

[bib45] Rahman, F.A., N. Aziz, and D. Coverley. 2010. Differential detection of alternatively spliced variants of Ciz1 in normal and cancer cells using a custom exon-junction microarray. BMC Cancer. 10:482. 10.1186/1471-2407-10-482 20831784 PMC2945943

[bib46] Ridings-Figueroa, R., E.R. Stewart, T.B. Nesterova, H. Coker, G. Pintacuda, J. Godwin, R. Wilson, A. Haslam, F. Lilley, R. Ruigrok, . 2017. The nuclear matrix protein CIZ1 facilitates localization of Xist RNA to the inactive X-chromosome territory. Genes Dev. 31:876–888. 10.1101/gad.295907.117 28546514 PMC5458755

[bib47] Rodermund, L., H. Coker, R. Oldenkamp, G. Wei, J. Bowness, B. Rajkumar, T. Nesterova, D.M. Susano Pinto, L. Schermelleh, and N. Brockdorff. 2021. Time-resolved structured illumination microscopy reveals key principles of Xist RNA spreading. Science. 372:eabe7500. 10.1126/science.abe7500 34112668

[bib48] Schindelin, J., I. Arganda-Carreras, E. Frise, V. Kaynig, M. Longair, T. Pietzsch, S. Preibisch, C. Rueden, S. Saalfeld, B. Schmid, . 2012. Fiji: An open-source platform for biological-image analysis. Nat. Methods. 9:676–682. 10.1038/nmeth.2019 22743772 PMC3855844

[bib49] Sirchia, S.M., S. Tabano, L. Monti, M.P. Recalcati, M. Gariboldi, F.R. Grati, G. Porta, P. Finelli, P. Radice, and M. Miozzo. 2009. Misbehaviour of XIST RNA in breast cancer cells. PLoS One. 4:e5559. 10.1371/journal.pone.0005559 19440381 PMC2679222

[bib50] Sofi, S., L. Williamson, G.L. Turvey, C. Scoynes, C. Hirst, J. Godwin, N. Brockdorff, J. Ainscough, and D. Coverley. 2022. Prion-like domains drive CIZ1 assembly formation at the inactive X chromosome. J. Cell Biol. 221:e202103185. 10.1083/jcb.202103185 35289833 PMC8927971

[bib51] Soneson, C., M.I. Love, and M.D. Robinson. 2015. Differential analyses for RNA-seq: Transcript-level estimates improve gene-level inferences. F1000 Res. 4:1521. 10.12688/f1000research.7563.1 PMC4712774 26925227

[bib52] Stewart, E.R., R.M.L. Turner, K. Newling, R. Ridings-Figueroa, V. Scott, P.D. Ashton, J.F.X. Ainscough, and D. Coverley. 2019. Maintenance of epigenetic landscape requires CIZ1 and is corrupted in differentiated fibroblasts in long-term culture. Nat. Commun. 10:460. 10.1038/s41467-018-08072-2 30692537 PMC6484225

[bib53] Subramanian, A., P. Tamayo, V.K. Mootha, S. Mukherjee, B.L. Ebert, M.A. Gillette, A. Paulovich, S.L. Pomeroy, T.R. Golub, E.S. Lander, and J.P. Mesirov. 2005. Gene set enrichment analysis: A knowledge-based approach for interpreting genome-wide expression profiles. Proc. Natl. Acad. Sci. USA. 102:15545–15550. 10.1073/pnas.0506580102 16199517 PMC1239896

[bib54] Sunwoo, H., D. Colognori, J.E. Froberg, Y. Jeon, and J.T. Lee. 2017. Repeat E anchors Xist RNA to the inactive X chromosomal compartment through CDKN1A-interacting protein (CIZ1). Proc. Natl. Acad. Sci. USA. 114:10654–10659. 10.1073/pnas.1711206114 28923964 PMC5635913

[bib55] Swarts, D.R.A., E.R. Stewart, G.S. Higgins, and D. Coverley. 2018. CIZ1-F, an alternatively spliced variant of the DNA replication protein CIZ1 with distinct expression and localisation, is overrepresented in early stage common solid tumours. Cell Cycle. 17:2268–2283. 10.1080/15384101.2018.1526600 30280956 PMC6226236

[bib56] Tang, Z., C. Li, B. Kang, G. Gao, C. Li, and Z. Zhang. 2017. GEPIA: A web server for cancer and normal gene expression profiling and interactive analyses. Nucleic Acids Res. 45:W98–W102. 10.1093/nar/gkx247 28407145 PMC5570223

[bib57] Tate, J.G., S. Bamford, H.C. Jubb, Z. Sondka, D.M. Beare, N. Bindal, H. Boutselakis, C.G. Cole, C. Creatore, E. Dawson, . 2019. COSMIC: The catalogue of somatic mutations in cancer. Nucleic Acids Res. 47:D941–D947. 10.1093/nar/gky1015 30371878 PMC6323903

[bib58] Thennavan, A., F. Beca, Y. Xia, S.G. Recio, K. Allison, L.C. Collins, G.M. Tse, Y.Y. Chen, S.J. Schnitt, K.A. Hoadley, . 2021. Molecular analysis of TCGA breast cancer histologic types. Cell Genom. 1:100067. 10.1016/j.xgen.2021.100067 35465400 PMC9028992

[bib59] Topa, H., C. Benoit-Pilven, T. Tukiainen, and O. Pietiläinen. 2024. X-chromosome inactivation in human iPSCs provides insight into X-regulated gene expression in autosomes. Genome Biol. 25:144. 10.1186/s13059-024-03286-8 38822397 PMC11143737

[bib60] Turvey, G.L., E.L. de Alba, E. Stewart, L. Byrom, H. Cook, S. Sofi, A. Alalti, J.F.-X. Ainscough, A. Mason, A.A. Antson, and D. Coverley. 2023. Dominant CIZ1 fragments drive epigenetic instability and are expressed in early stage cancers. bioRxiv. 10.1101/2023.09.22.558821 (Preprint posted September 22, 2023).

[bib61] Valledor, M., M. Byron, B. Dumas, D.M. Carone, L.L. Hall, and J.B. Lawrence. 2023. Early chromosome condensation by XIST builds A-repeat RNA density that facilitates gene silencing. Cell Rep. 42:112686. 10.1016/j.celrep.2023.112686 37384527 PMC10461597

[bib62] Veiga, D.F.T., A. Nesta, Y. Zhao, A. Deslattes Mays, R. Huynh, R. Rossi, T.C. Wu, K. Palucka, O. Anczukow, C.R. Beck, and J. Banchereau. 2022. A comprehensive long-read isoform analysis platform and sequencing resource for breast cancer. Sci. Adv. 8:eabg6711. 10.1126/sciadv.abg6711 35044822 PMC8769553

[bib63] Xiao, J., S.R. Vemula, and M.S. LeDoux. 2014a. Recent advances in the genetics of dystonia. Curr. Neurol. Neurosci. Rep. 14:462. 10.1007/s11910-014-0462-8 24952478 PMC4886715

[bib64] Xiao, Y., T.H. Hsiao, U. Suresh, H.I. Chen, X. Wu, S.E. Wolf, and Y. Chen. 2014b. A novel significance score for gene selection and ranking. Bioinformatics. 30:801–807. 10.1093/bioinformatics/btr671 22321699 PMC3957066

[bib65] You, J.S., and P.A. Jones. 2012. Cancer genetics and epigenetics: Two sides of the same coin? Cancer Cell. 22:9–20. 10.1016/j.ccr.2012.06.008 22789535 PMC3396881

