# Supplementary material for: Epigenetic deprogramming by disruption of CIZ1-RNA nuclear assemblies in early-stage breast cancers
Source: J Cell Biol. 2025 Mar 11;224(5):e202409123. doi: 10.1083/jcb.202409123 (PMC11895699; doi:10.1083/jcb.202409123)
Supplement: SourceData F4 — is the source file for Fig. 4. [file jcb_202409123_sourcedataf4.pdf]

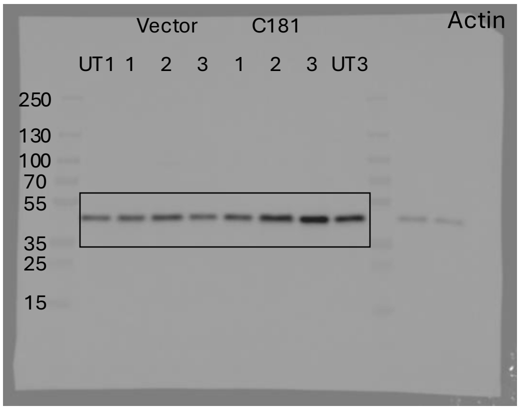


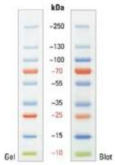

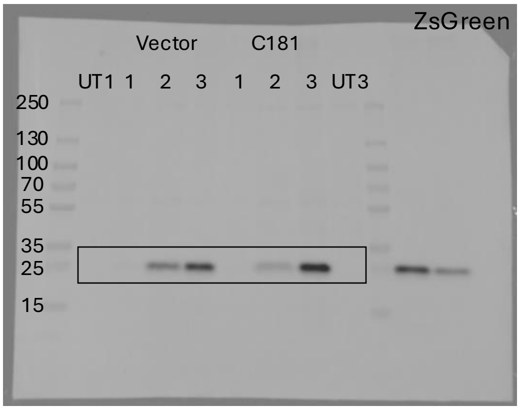


C181 (E17 Ab, reprobe of actin blot)

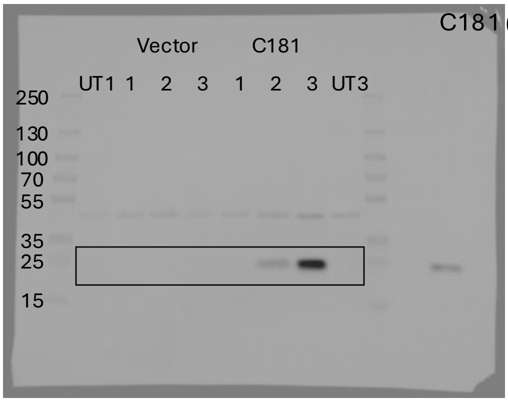


This Source Data consists of fully uncropped and unprocessed images for each blot displayed in the figures. The lanes of the blots are labelled as they are in the associated figure, and the place where cropping was applied is marked with a box. Molecular weight standards are labelled.
